# Dynamics in the Vicinity of the Stable Halo Orbits

**DOI:** 10.1007/s40295-023-00379-7

**Published:** 2023-06-27

**Authors:** David Lujan, Daniel J. Scheeres

**Affiliations:** Aerospace Engineering Sciences, University of Colorado Boulder, 3775 Discovery Dr., Boulder, 80309 Colorado USA

**Keywords:** Quasi-periodic orbits, Invariant tori, Halo orbits, Orbit families

## Abstract

This work presents a study of the dynamics in the vicinity of the stable ***L***_***2***_ halo orbits in the Earth-Moon system of the circular restricted three-body problem. These solutions include partially elliptic, partially hyperbolic, and elliptic quasi-halo orbits. The first two types of orbits are 2-dimensional quasi-periodic tori, whereas the elliptic orbits are 3-dimensional quasi-periodic tori. Motivated by the Lunar Gateway, this work computes these orbits to explore the 3-parameter family of solutions lying in the vicinity of the stable halo orbits. An algorithm is presented to quantify the size of the invariant surfaces which gives perspective on the size of the orbits. A stability bifurcation is detected where the partially elliptic tori become partially hyperbolic. A nonlinear behavior of the Jacobi constant is observed which differs from the behavior of the quasi-halo orbits emanating from the unstable halo orbits which makeup the majority of the quasi-halo family. Uses of the orbits in the vicinity of the stable ***L***_***2***_ halo orbits are identified, and the results highlight characteristics and structure of the family to broaden the understanding of the dynamical structure of the circular restricted three-body problem.

## Introduction

In the circular restricted three-body problem (CR3BP), an autonomous Hamiltonian dynamical system with 3 degrees of freedom, there are special solutions equivalent to 0-, 1-, 2-, and 3-dimensional tori. The 0-d tori are equilibrium points, 1-d tori are periodic orbits (POs) while 2- and 3-d quasi-periodic tori (QPTs) are quasi-periodic orbits. A quasi-periodic orbit (QPO) is an orbit diffeomorphic to a QPT. In other words, a particle on such an orbit never repeats its state and the resulting trajectory forms a surface or volume in phase space after infinite time. QPOs are higher dimensional objects than POs and are conceptually and computationally more challenging to compute.

Benefits of utilizing QPOs over POs include a higher abundance of orbits. In the CR3BP, POs lie in 1-parameter families while QPOs lie in 2- and 3-parameter families. Additionally, for unstable orbits, there are more options to utilize invariant manifolds. The combination of the higher dimension of QPOs with the higher dimension of their families produce larger dimensional hyperbolic bundles compared to those of families of POs. Lastly, POs generically become QPOs when transitioned into higher fidelity dynamical models, so the ability to compute families of QPOs is important for study and mission design.

In the astrodynamics literature there is work that compute tori of dimension greater than two. Jorba and Olmedo compute tori of dimension three in the CR3BP Sun-Jupiter system with the addition of the gravitational influence of Saturn, Uranus, Neptune, and Earth [1]. The orbit they found is the resulting motion of the fifth Lagrange point of the original Sun-Jupiter system. In their approach, knowledge of all the torus frequencies is a requisite to the computation of the tori. Baresi and Scheeres compute a family of 3-d QPOs in the time periodic system of asteroid 4179 Toutatis for the purposes of small body exploration [2]. Due to the time dependent nature of the dynamics one of the frequencies is fixed to the frequency of rotation of the asteroid. This, in turn, results in a 2-parameter family of 3-d tori. In McCarthy and Howell a single 1-parameter family of elliptic quasi-halos in the CR3BP are computed where the longitudinal frequency and Jacobi constant are fixed among the family members [3]. This work shows their ability to compute 3-tori, but no analysis is done on any family of 3-tori. Gimeno et al. in [4] extend the methods of Jorba and Olmedo [1] to compute the resulting motion of the Lagrange points in the CR3BP Earth-Moon system under the influence of up to five perturbing frequencies. This results in tori up to dimension five. Gabern et al. also compute tori up to dimension five in the Tricircular Coherent Problem [5]. In these last two references continuation is not used, meaning only singular orbits are computed. Moreover, a-priori knowledge of the frequencies is necessary in these computations.

In the CR3BP the families of periodic halo orbits have been studied in the well-known papers of Breakwell and Brown [6], Howell [7], and Howell and Breakwell [8], while the 2-parameter families of quasi-halo orbits have been studied in Gómez and Mondelo [9], Haro and Mondelo [10], and Lujan and Scheeres [11]. The orbits in these studies are primarily partially hyperbolic, however in the *L*_*2*_ family of halo orbits there exists a stable region of elliptic orbits. In the vicinity of the elliptic halo orbits are 2-parameter families of partially elliptic quasi-halo orbits [11] and a 3-parameter family of elliptic quasi-halo orbits.

More work exists on the topic of computing QPOs in astrodynamics, however it seems no attempts have been made to study a 3-parameter family, therefore it is of academic interest to study a 3-parameter family of orbits. Due to the interest in the Earth-Moon *L*_*2*_ halo orbits arising from the Artemis program the dynamics in the vicinity of the stable halo orbits in the Earth-Moon system of the CR3BP have been chosen for study here. Studying this family will increase the body of literature on solutions in the CR3BP, deepening the understanding of this fundamental dynamical system, while bringing into light a lesser-known family of solutions.

The remainder of the paper is structured as follows: Sect. 2 covers the dynamical system in which the orbits are computed. Section 3 provides background introducing QPTs and their connection to orbits in astrodynamics, the monodromy matrix, and the periodic halo orbits. Section 4 goes over the research methods used in this work. Section 5 presents and discusses the results while Sect. 6 concludes the paper.

## Circular Restricted Three-Body Problem

### Equations of Motion

The dynamical system of study here is the CR3BP. It is the study of motion of a massless particle under the gravitational forces of two massive bodies $P_1$ and $P_2$ in circular orbits about their common center of mass with $m_1\geq m_2$. The dynamics are stated in a rotating frame such that the *x*-axis points from $P_1$ to $P_2$, the *z*-axis is aligned with the angular momentum vector, and the *y*-axis completes the right-handed coordinate system. The equations are written in a non-dimensional (ND) form where the distance between $P_1$ and $P_2$ and the mean motion are equal to one. The dimensionless mass parameter is defined as $\mu =m_2/(m_1+m_2)$. The equations of motion take the following form

1 $$\begin{aligned} \ddot{x}&= x + 2\dot{y} - \frac{(1-\mu )(x+\mu )}{r_1^3} - \frac{\mu (x-1+\mu )}{r_2^3}\nonumber \\ \ddot{y}&= y - 2\dot{x} - \frac{(1-\mu )y}{r_1^3} - \frac{\mu y}{r_2^3}\nonumber \\ \ddot{z}&= -\frac{(1-\mu )z}{r_1^3} - \frac{\mu z}{r_2^3} \end{aligned}$$

where $r_1=\sqrt{(x+\mu )^2+y^2+z^2}$ is the distance to $P_1$ and $r_2=\sqrt{(x-1+\mu )^2+y^2+z^2}$ is the distance to $P_2$. The value of $\mu$ used is 0.012153599037880 which describes the Earth-Moon system.

### Jacobi Constant

System (1) admits one integral of motion called the Jacobi constant which is an energy-like quantity that determines which areas of phase space are accessible and which other orbits can be reached without changing energy levels. Equation (2) defines the Jacobi constant given a state vector ${\varvec{x}}$ in non-dimensional units. In the given form a lower value of *J* correlates to higher energies at which more areas of space can be accessed.

2 $$\begin{aligned} J({\varvec{x}}) = 2\left( \frac{1-\mu }{r_1}+\frac{\mu }{r_2}\right) + x^2 + y^2 - (\dot{x}^2+\dot{y}^2+\dot{z}^2) \end{aligned}$$

### Solutions

System (1) has five equilibrium points labelled sequentially $L_1$ through $L_5$. For the value of $\mu$ used here $L_1$ through $L_3$ are of type *center* x *center* x *saddle* while $L_4$ and $L_5$ are of type *center* x *center* x *center*. This work is focused on QPOs about the $L_2$ libration point. One of the center manifolds of $L_2$ produces oscillations in the *x*-*y* plane giving rise to the planar Lyapunov orbits. This family of orbits begins as type *center* x *center* x *saddle* and eventually bifurcates to *center* x *saddle* x *saddle*. At this bifurcation point a new family of periodic orbits, the family of halo orbits, is born. This family and the resulting bifurcated family of quasi-halo orbits will be described in Sect. 3.3.

## Background

### Quasi-Periodic Invariant Tori and Orbits

A quasi-periodic invariant torus is the closure of a quasi-periodic trajectory lying on the surface of a *n*-dimensional torus $\mathbb {T}^n$ satisfying the dynamics

3 $$\begin{aligned} {\dot{\theta }}_i = \omega _i, \hspace{.15in} i=0,1,\dots ,n-1, \end{aligned}$$

where $\omega _i$ are constants called the frequencies of the torus and are incommensurate with each other. Each dimension of the torus has an associated amplitude $A_i$ and rotation number $\rho _i=\omega _i T$ where *T* is called the stroboscopic time. In this work *T* is derived from $\omega _0$, and in this case $\rho _0 = 2\pi$.

**Fig. 1:**
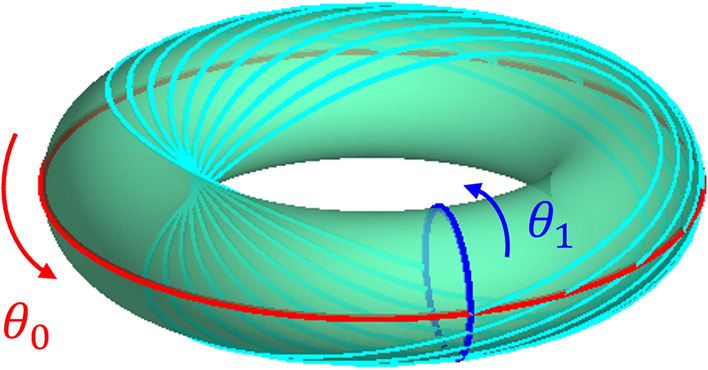
Example of a 2-d QPT

Figure 1 shows an example of a 2-d QPT with coordinates $\theta _0$ and $\theta _1$ and a partial trajectory. The trajectory has incommensurate frequencies $\omega _0$ and $\omega _1$. When the trajectory has flowed to integer multiples of *T* the angular displacements of the trajectory compared to its starting position have rotated by corresponding integer multiples of the rotation numbers on the torus. This behavior is seen in the figure when the trajectory crosses the $\theta _1$ circle. As the trajectory evolves in time it will fully wrap around the surface of the torus without ever repeating its state.

Many orbits in Hamiltonian systems, such as the CR3BP, are diffeomorphic to QPTs. Equilibrium points are 0-d QPTs, POs are 1-d QPTs, and QPOs are QPTs of dimension 2 and higher. In autonomous Hamiltonian systems with *n* degrees of freedom there exist tori with maximal dimension of *n*, so in the CR3BP the maximal dimension of tori is 3. These maximal dimensional tori must be elliptic since all the available degrees of freedom are being consumed by center manifolds. Additionally, in autonomous Hamiltonian systems, POs makeup continuous families ([12] Chapter 9) while QPOs makeup Cantorian families [13, 14]. A Cantorian family is nearly continuous except where the frequencies are commensurable. From here on out when referring to a dynamical system it is implied that the system is an autonomous Hamiltonian system.

Under certain conditions, which will be discussed in the next Sect. 3.2, a family of QPOs emanate from a single PO. This means the dimension of the family of QPOs is larger than the dimension of the family of POs. An implication of this is that families of POs need only one parameter to specify a member of the family and are graphically depicted as a line. However, families of QPOs need at least 2 parameters to specify a member meaning they are graphically depicted as a surface or a volume. Additionally, the higher dimension of the family adds a computational complexity when finding members to represent the family as it is not trivial to move about the solution space and many more representative members are needed to represent the family.

### The Monodromy Matrix of Autonomous Hamiltonian Systems

One way the existence of QPOs in a dynamical system are identified are from the monodromy matrix. This is the state transition matrix corresponding to a PO and evaluated at the period of that PO. It is a symplectic matrix, therefore the eigenvalues come in reciprocal pairs ( [15] Appendix D). The eigenvalues of the monodromy matrix indicate the types of linear behavior about the PO while the eigenvectors give the directions tangent to the nonlinear invariant manifolds emanating from the orbit ( [16] Chapter 4).

Eigenvalues on the unit circle indicate center manifolds and otherwise indicate hyperbolic manifolds. The existence of non-unity eigenvalues on the unit circle indicate there is a center manifold normal to the PO. If the frequency of this center manifold is incommensurate with the base frequency of the PO then a 2-parameter family of QPOs foliate this subspace spanned by the family of POs. However, if these frequencies are commensurate then the center subspace is foliated by a 1-parameter family of POs.

### The Halo Orbit Family

The family of halo orbits is primarily of type *center* x *center* x *saddle*, however there is a region at which the family becomes of type *center* x *center* x *center*. In this stable region there are families of 2- and 3-d QPTs. The 2-parameter families of quasi-halos contain both partially hyperbolic and partially elliptic orbits [11] while the 3-parameter family of quasi-halos are purely elliptic orbits.

**Fig. 2:**
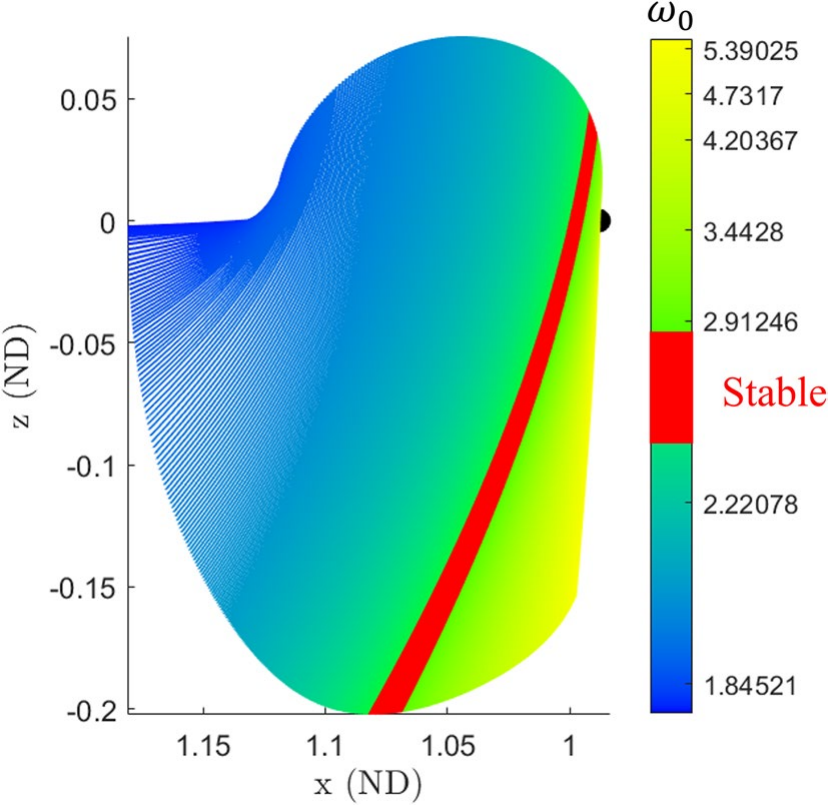
$L_2$ halo orbit family in the Earth-Moon system

Figure 2 shows an *x*-*z* view of the $L_2$ family of halo orbits in position space colored according to their base frequency $\omega _0$. In the figure the stable orbits are a solid color to distinguish it from the unstable orbits. The Moon is plotted to scale as a sphere to serve as a reference for the size and location of the orbits.

**Fig. 3:**
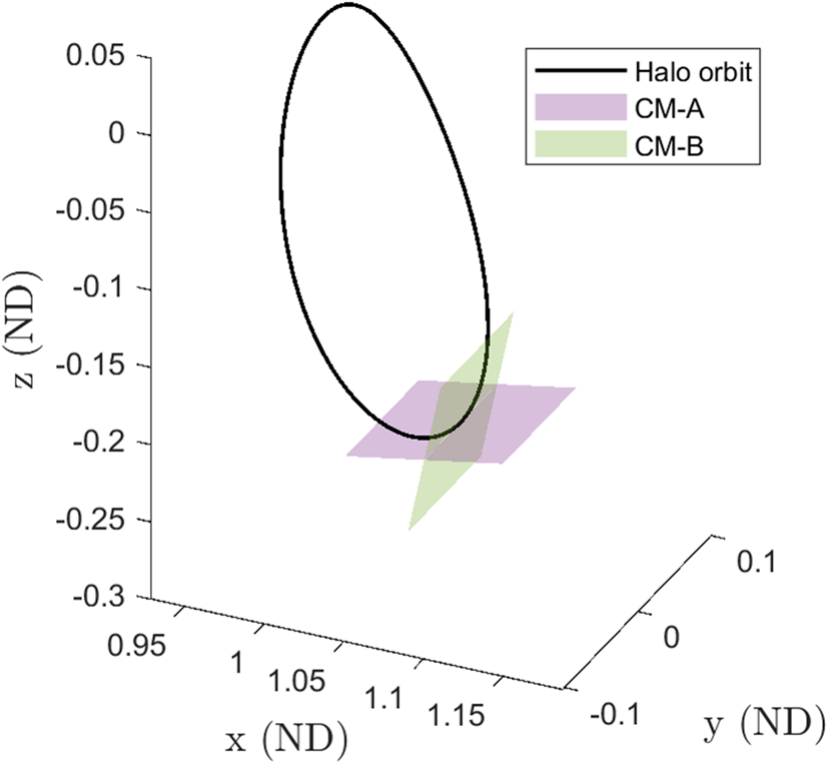
A stable halo orbit with the linear center manifolds from its monodromy matrix

**Fig. 4:**
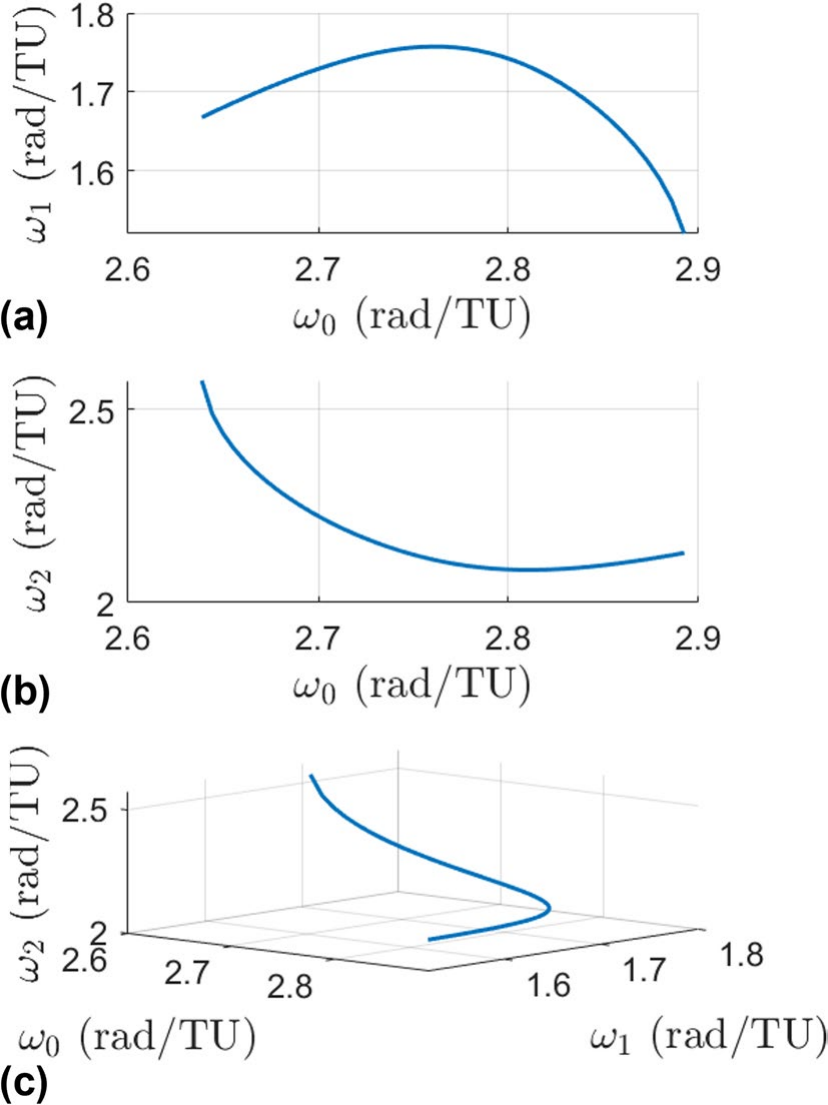
Frequencies of the stable $L_2$ halo orbit family and the frequencies of their normal center subspaces

Halo orbits in the stable region have a base frequency $\omega _0$ (defined as $2\pi /T$ where *T* is the period of the orbit) and two additional frequencies $\omega _1$ and $\omega _2$ that arise from the normal linear center manifolds. The frequency $\omega _0$ dictates the frequency of moving along the PO, while the frequencies $\omega _1$ and $\omega _2$ dictate the frequencies of oscillations about the halo orbit. The set $(\omega _0,\omega _1,\omega _2)$ thus approximate the frequencies of small amplitude quasi-halos in the vicinity of the halo orbit. Since there are two normal center manifolds they are referred to as CM-A and CM-B to distinguish each other. Figure 3 shows a stable halo orbit with these two additional linear center manifolds extending from a single point along the orbit. CM-A has frequency $\omega _1$ while CM-B has frequency $\omega _2$. Figure 4 shows these linear frequencies for the stable region. The top plot shows $\omega _0$ versus $\omega _1$, the middle plot shows $\omega _0$ versus $\omega _2$, and the bottom plot shows a view of the frequencies in a 3-d space. The line seen in these plots is called the halo line because it is a line that represents data about the halo orbit family.

## Solution Method

The method to compute subsets of the 2- and 3-parameter family of quasi-halo orbits rely on a single-parameter continuation method called GMOS [17] (Gómez—Mondelo—Olikara—Scheeres) which is adapted with various parametric constraints. A sequence of solutions from GMOS is called a branch of solutions as it is a 1-dimensional slice through the higher-dimensional solution space. Each branch is initialized from a linear approximation of a 2- or 3-torus emanating from a stable halo orbit. There are four different types of branches that are initialized from each halo orbit. The first type contains solutions of 2-d QPOs initialized by stepping onto CM-A, the second contains solutions of 2-d QPOs initialized by stepping onto CM-B. Each of these orbit types have two frequencies, however the frequency pair for the second branch type is $(\omega _0,\omega _2)$ in order to make the labelling of the frequencies consistent with which center manifold they correspond to. The third branch type contains solutions of 3-d QPOs initialized by stepping onto both center manifolds and holding $\omega _2$ constant. The last branch type contains solutions of 3-d QPOs which hold $\omega _1$ constant. The four branch types are computed from a span of elliptic halo orbits and pieced together to form four approximate 2-parameter families.

The first branch type is called the "constant $\omega _2$ 2-d" branch since $\omega _1$ changes throughout the branch while $\omega _2$ is held constant, which effectively mutes motion in CM-B. Likewise the second branch type is called the "constant $\omega _1$ 2-d" branch. The third branch is called the "constant $\omega _2$ 3-d" branch, and lastly the fourth branch is called the "constant $\omega _1$ 3-d" branch.

This section is broken into three main subsections. Section 4.1 describes how to seed an initial guess of 2-d and 3-d QPOs from a PO, Sect. 4.2 describes the governing constraint equations of the QPO computational algorithm GMOS, Sect. 4.3 describes the methodology to compute each branch of solutions mentioned previously, and Sect. 4.4 goes over the method to compute the amplitudes of an invariant surface.

### Initial Guess

**Fig. 5:**
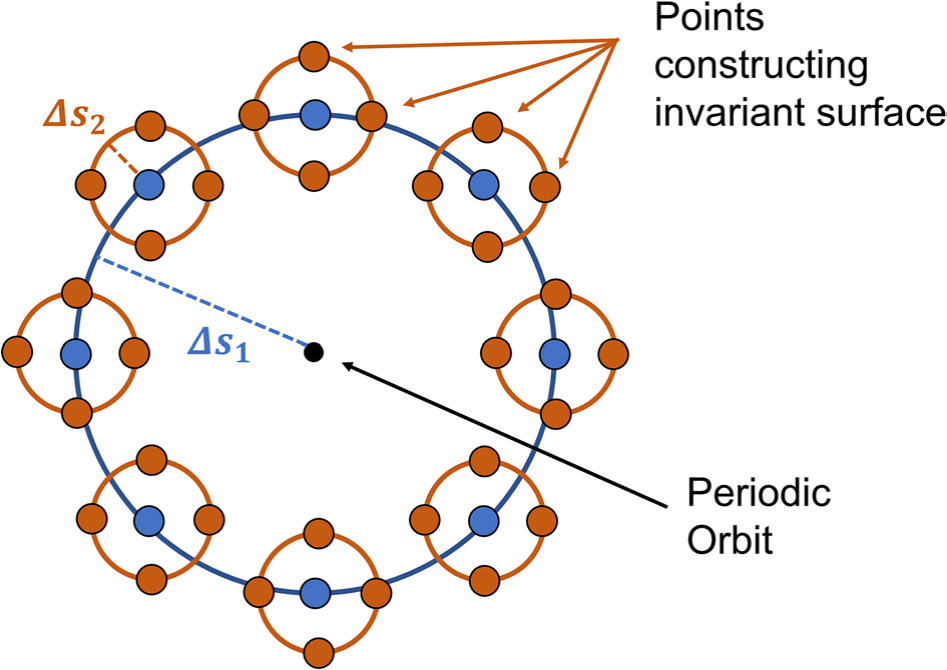
Representation of an invariant surface of a 3-d QPT

A guess of an *n*-d quasi-periodic orbit is initialized from the center eigenspaces of the monodromy matrix of a periodic orbit ${\varvec{y}}:\mathbb {T}\rightarrow \mathbb {R}^d$. This initial guess is an approximation of an invariant surface ${\varvec{X}}:\mathbb {T}^{n-1}\rightarrow \mathbb {R}^d$ with associated stroboscopic time *T* and rotation vector ${\varvec{\rho }}$. Suppose the periodic orbit with period *T* has a monodromy matrix with $n-1$ center subspaces, then there are $n-1$ center eigenvalues $\lambda _{j}$ along with their complex conjugates. Additionally, there are $n-1$ center eigenvectors ${\varvec{u}}_j$ along with their complex conjugates that are tangent to the nonlinear center manifolds. The initial guess of the invariant surface is constructed according to Eq. (4). An invariant surface ${\varvec{X}}(\theta _1,\dots ,\theta _{n-1})$ is constructed from the ${\varvec{u}}_j$ and chosen step sizes $\Delta s_j$ (Eq. (4). The $\Delta s_j$ represent the amplitudes of steps taken onto each center subspace in order to produce small oscillations in that plane. To get an accurate initial guess $\Delta s_1$ and $\Delta s_2$ should be on the order of 1e-6. A representation of the invariant surface of a 3-d QPO is given in Fig. 5. It is noted that the points lying on the large central curve are also thought of as the points constructing the invariant curve of a 2-d QPO. The initial guess of the stroboscopic time of the QPO is taken to be the period of the PO. The initial guess of the rotation numbers $\rho _j$ is taken to be the argument of each $\lambda _j$. Note that it is valid to use the argument of the complex conjugates as well.

4 $$\begin{aligned} {\varvec{X}}(\theta _1,\dots ,\theta _{n-1})={\varvec{y}}(0) + \sum _{j=1}^{n-1}\Delta s_j (\text {Re}({\varvec{u}}_j )\cos {\theta _j} - \text {Im}({\varvec{u}}_j )\sin {\theta _j}) \end{aligned}$$

### GMOS

GMOS is a predictor-corrector method inside a single-parameter continuation algorithm formulated in a single-shooting, multiple-shooting, and collocation method [18]. It was first developed by Gómez and Mondelo in [9] and modified by Olikara and Scheeres [17]. In the way GMOS is typically used only 1-parameter subsets of *p*-parameter families of *n*-tori can be computed. This is done by defining a set of equations that define an implicit 1-dimensional manifold. This set of equations constitutes a quasi-periodicity constraint (Eq. (5)), *n* phase constraints (Eqs. (6) and (7)), and *p* parametric constraints; one of which is a pseudo-arclength constraint (Eq. (8)). The use of Eq. (8) allows for single-parameter continuation and enforces the step size between the previous solution and current solution (see [19] Chapter 4). See [2, 3, 11, 20] for more information on GMOS and some use cases.

The independent variables for this algorithm are ${\varvec{X}}=[{\varvec{x}}_1^T,...,{\varvec{x}}_j^T,...]^T$, a vector of discrete points ${\varvec{x}}_j$ in phase space that partition $\mathbb {T}^n$ into evenly spaced intervals, *T*, a stroboscopic time, and ${\varvec{\rho }}$, a vector of rotation numbers. A Newton’s method is used to correct the QPO until the norm of the constraint vector is under the specified tolerance level. For the computation of *q*-parameter families of QPOs in autonomous Hamiltonian systems both *p* and *n* are equal to *q*, therefore *q* phase constraints are needed along with $q-1$ additional parametric constraints on top of the pseudo-arclength constraint.

5 $$\begin{aligned} R_{-\varvec{\rho} }\phi _T ({\varvec{X}}) - {\varvec{X}}&= {\varvec{0}}\end{aligned}$$

6 $$\begin{aligned} \Bigl <{\varvec{X}}-\tilde{{\varvec{X}}},\frac{{\partial {\varvec{X}}}}{\partial \theta _{0}}\Bigr >&= 0\end{aligned}$$

7 $$\begin{aligned} \Bigl <{\varvec{X}},\frac{{\partial {\varvec{X}}}}{\partial \theta _{j}}\Bigr >&= 0,\hspace{.15in} j=1,2,\dots ,n-1\end{aligned}$$

8 $$\begin{aligned} \Bigl<{\varvec{X}}-\tilde{{\varvec{X}}},\tilde{{\varvec{X}}}'\Bigr> + (T-\tilde{T})\tilde{T}' + \Bigl <{\varvec{\rho }}-\tilde{{\varvec{\rho }}},\tilde{{\varvec{\rho }}}'\Bigr > - \delta s&= 0\end{aligned}$$

9 $$\begin{aligned} s_i ({\varvec{X}},T,{\varvec{\rho }})&= 0, \hspace{.15in}i=1,2,\dots ,p-1 \end{aligned}$$

In the above equations the tilde represents the previous values of the underlying quantity and the prime represents the family tangent values. Both of these types of quantities are needed for pseudo-arclength continuation.

### Computing Subsets of the Family

To construct branches of solutions Eqs. (5-9) must all be considered, however there is a choice for the remaining parametric constraints. The choice of constraints provides freedom in choosing which characteristics are in common for the branch and which ones are different. In the computations here the stroboscopic time is always chosen to be consistent with the period of the underlying halo orbit $T^*$ (Eq. (10)). This constraint corresponds to keeping the base frequency $\omega _0$ constant among the branch.

10 $$\begin{aligned} T-T^* = 0 \end{aligned}$$

#### 2-d Quasi-Periodic Tori

For the computation of the branches of 2-d quasi-halo orbits both *n* and *p* are equal to two, so no extra parametric constraints are added to GMOS. The difference between the constant $\omega _2$ and constant $\omega _1$ branches is which center manifold the branch is initialized onto. Visually this represents taking a step onto either CM-A or CM-B from Fig. 3, and numerically this means letting either $\Delta s_1$ or $\Delta s_2$ be zero while the other is nonzero.

#### 3-d Quasi-Periodic Tori

For the computation of the branches of 3-d quasi-halo orbits both *n* and *p* are equal to three, so one additional parametric constraint is added to GMOS. The 3-d quasi-halo orbits are initialized onto both CM-A and CM-B with nonzero $\Delta s$’s, so the use of the additional parametric constraint differentiates between the two types of branches. The constant $\omega _2$ 3-d branch uses Eq. (11) while the constant $\omega _1$ branch uses Eq. (12). The starred variables in the equations below represent fixed values which are determined from the underlying halo orbit and its monodromy matrix.

11 $$\begin{aligned} \frac{\rho _1}{T} - \frac{\rho _1^*}{T^*}&= 0\end{aligned}$$

12 $$\begin{aligned} \frac{\rho _2}{T} - \frac{\rho _2^*}{T^*}&= 0 \end{aligned}$$

The use of Eq. (10) with either Eqs. (11) or (12) constructs branches, such that, when plotted in a 3-dimensional frequency space according to the frequencies of the orbits, the branches make vertical or horizontal lines respectively.

### Amplitude Computation

A heuristic method is presented to define and compute the amplitudes of an *n*-dimensional discretized invariant surface ${\varvec{X}}$. First, given a set of integers $(N_{1},\dots ,N_{k})\in \mathbb {N}^k$ that form an evenly spaced grid over $\mathbb {T}^k$ define the multi-index ${\varvec{j}}=(j_{1},\dots ,j_{k})$ that belongs to the set $J_k\equiv \{{\varvec{j}}\in \mathbb {Z}^k|0\le j_i<N_i\hspace{.1in}\text {for}\hspace{.1in}i=1,\ldots ,k\}$. Then define the vector of angles ${\varvec{\theta }}_{{\varvec{j}}} = (\theta _{j_1},\ldots ,\theta _{j_k}) = 2\pi \left( j_1/N_1,\ldots ,j_k/N_k\right) \in \mathbb {T}^k$. Then ${\varvec{X}}({\varvec{\theta }}_{{\varvec{j}}})$ is a single point on the invariant surface and the collection of points representing a *k*-dimensional invariant surface is ${\varvec{X}}=\{{\varvec{X}}({\varvec{\theta }}_{{\varvec{j}}})|{\varvec{j}}\in J_k\}$. The notation ${\varvec{Y}}_{{\varvec{j}}}({\varvec{\theta }}_{k})=\{{\varvec{X}}({\varvec{\theta }}_{{\varvec{j}}},\theta _{k,l})|l=0,\ldots ,N_{k}-1\}$ represents a curve along the $k^{th}$ dimension of the invariant surface with fixed angles ${\varvec{\theta }}_{{\varvec{j}}}$ where ${\varvec{j}}\in J_{k-1}$ and ${\varvec{Y}}_{{\varvec{j}}}(\theta _{k,i})$ is a single point on the curve at $\theta _{k,i}=2\pi i/N_k$.

**Fig. 6:**
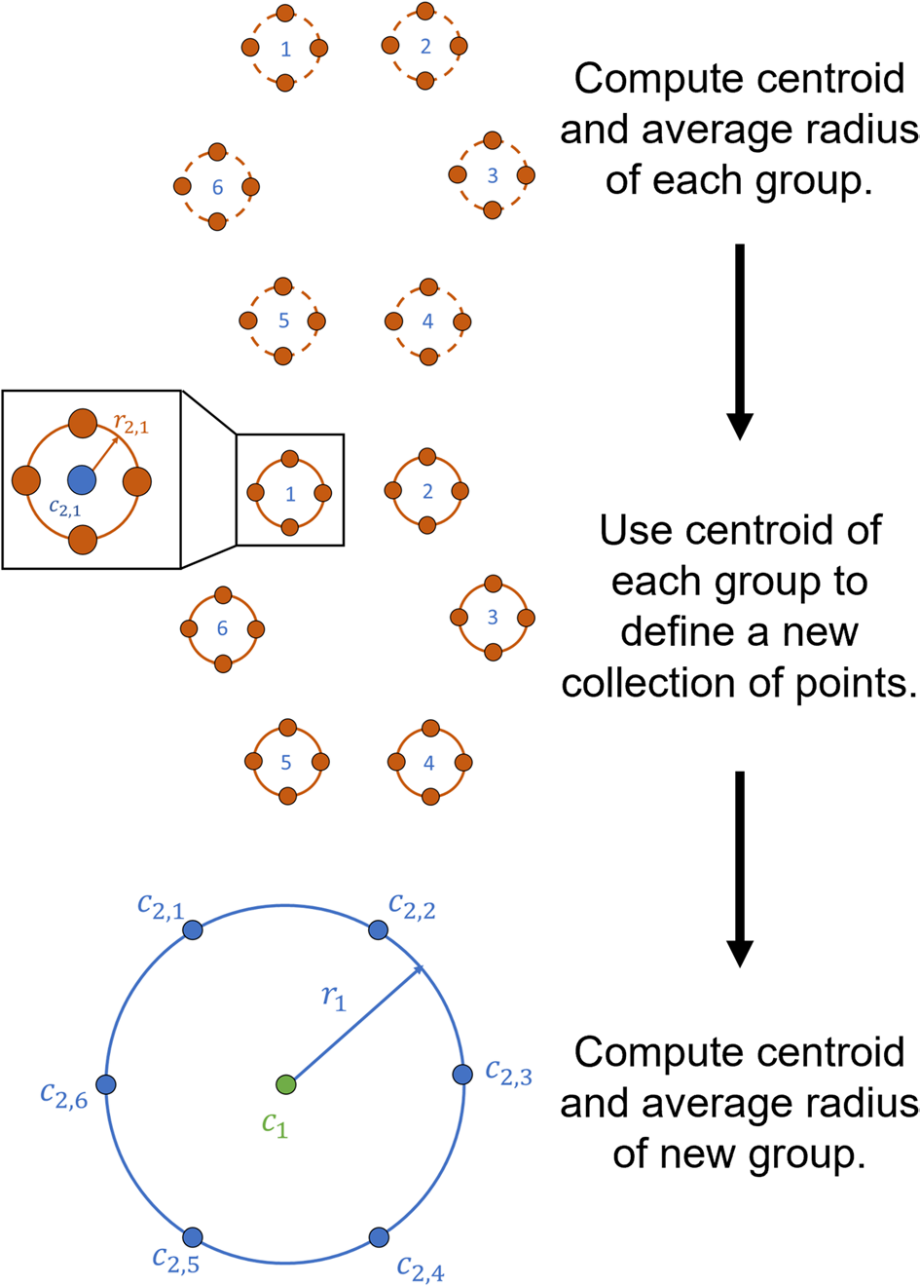
Example of computing amplitudes of an invariant surface with $n=2$, $N_1 = 6$, and $N_2 = 4$

The process to compute the amplitudes of an *n*-dimensional invariant surface ($k=n$) computes the amplitudes in reverse order starting with $A_n$. By freezing the first $n-1$ dimensions, a group of points is defined and forms a curve ${\varvec{Y}}_{{\varvec{j}}}({\varvec{\theta }}_{n})$. A centroid ${\varvec{c}}_{n,{\varvec{j}}}$ and average radius $r_{n,{\varvec{j}}}$ is computed from this curve and are recorded. The centroid and average radius are computed for each curve ${\varvec{Y}}_{{\varvec{j}}}({\varvec{\theta }}_{n})$ with ${\varvec{j}}\in J_{n-1}$. The amplitude $A_n$ is the average of the radius of each curve. The set of points *X* is redefined to be the set of centroids $\{{\varvec{c}}_{n,{\varvec{j}}}|{\varvec{j}}\in J_{n-1}\}$. This process has effectively reduced the dimension of the invariant surface by one by averaging out the last dimension. The process continues until all amplitudes have been computed. A 3-d QPT has an invariant surface that is a 2-dimensional, and as such, it has 2 amplitudes $A_1$ and $A_2$. The amplitude $A_0$ of the 3-d quasi-halo orbits correspond approximately to the sizes of the halo orbits, so these amplitudes have been omitted. Figure 6 depicts the process of computing the amplitudes for a 2-d invariant surface.
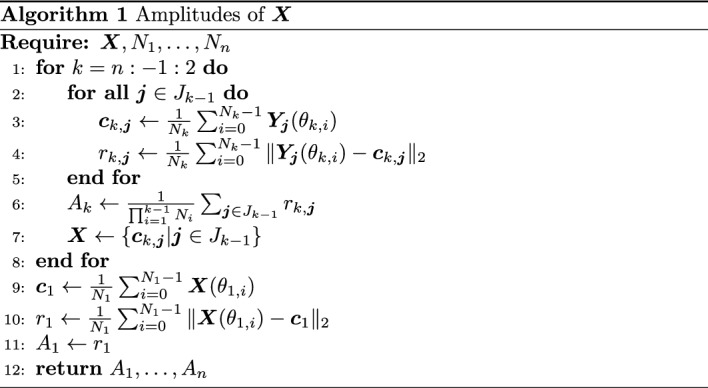

## Results

Each orbit has been computed with an error tolerance of 7e-11. The 2-d quasi-halo orbits use $N_1 = 111$ while the 3-d quasi-halos use $N_1 = 15$ and $N_2 = 11$. The reason for using 111 points to represent the invariant curves of the 2-d quasi-halos is because increasing the number of points increases the accuracy of the eigenvalues of the stability matrix [21]. The stability matrix of the 3-d quasi-halos is not necessary to compute as the orbits are inherently stable. The continuation of each branch is run until either: 1. The step size $\delta s$ has decreased to an allowable minimum step size. 2. The number of orbits computed reaches the maximum number of allowed orbits. The minimum step size for the 2-d quasi-halo orbits is taken to be 1e-5, while it is 6e-8 for the 3-d quasi-halo orbits. The maximum number of orbits is 120 for the 2-d quasi-halo orbits, while it is 100 for the 3-d quasi-halo orbits. Compiling the results from each 1-parameter branch gives four 2-d subsets of the solutions in the vicinity of the stable halo orbits.

### Frequencies

**Fig. 7:**
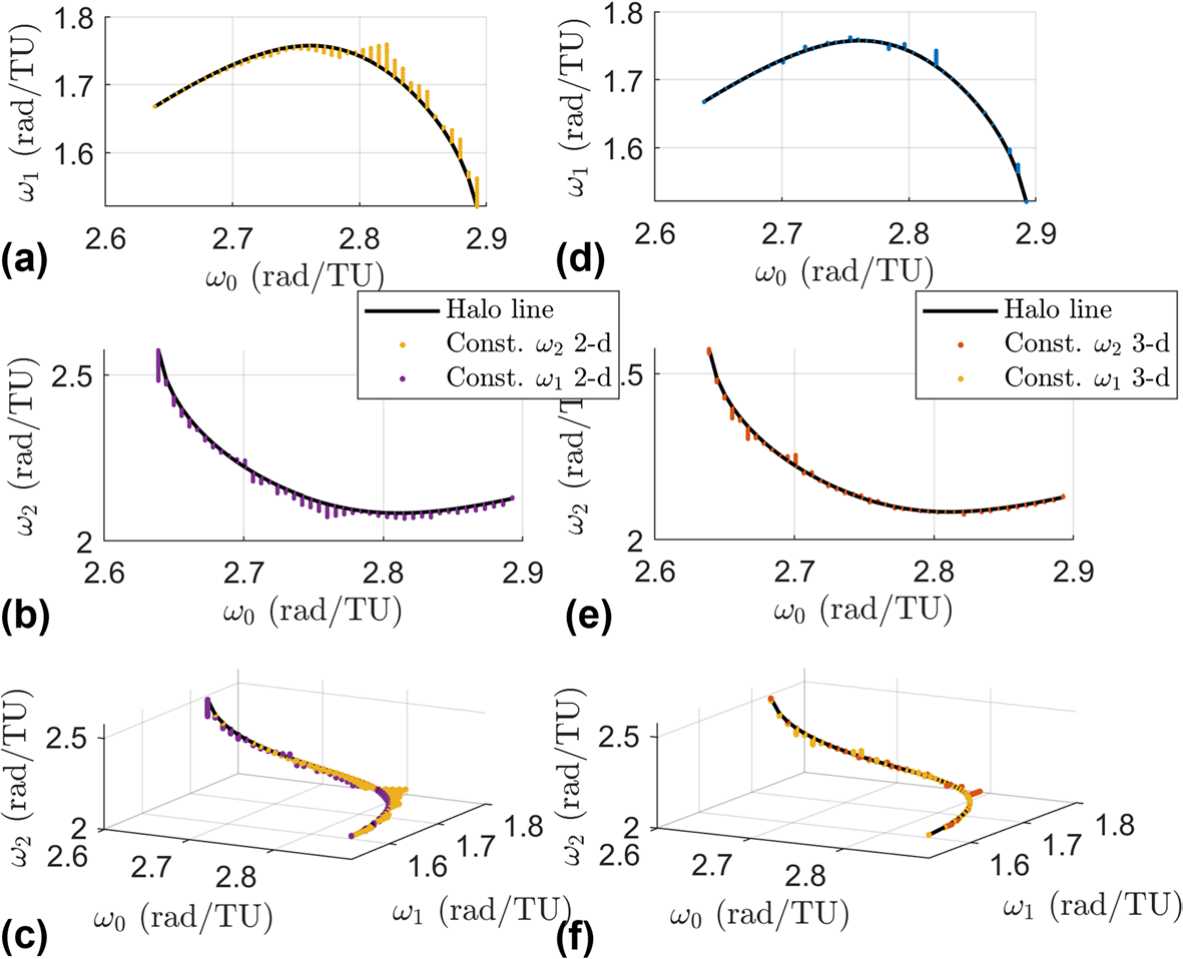
Frequencies of the four branches with different views

The frequencies of the orbits are in Fig. 7. Recall that each branch is a 1-parameter family of quasi-halo orbits grown from a halo orbit, so the plotted frequencies are lines extending from the frequencies of the halo orbits. Plot (a) of the figure shows the frequencies of the constant $\omega _2$ 2-d family, plot (d) is for the constant $\omega _2$ 3-d family, plot (b) is for the constant $\omega _1$ 2-d family, plot (e) is for the constant $\omega _1$ 3-d family, plot (c) is the combination of the constant $\omega _2$ 2-d and constant $\omega _1$ 2-d families in the three-dimensional frequency space, and plot (f) is the combination of the constant $\omega _2$ 3-d and constant $\omega _1$ 3-d families in the three-dimensional frequency space.

The families of 2-d tori fill more area in the frequency space than the families of 3-d tori, showing a wider variety in the rate of motion among the 2-d tori than the 3-d tori. This figure may lead one to believe that the measure of the 2-parameter families of 2-d tori is more than the measure of the 3-parameter family of the 3-tori, however this conclusion cannot be drawn. It is much easier to compute the 2-d tori than the 3-d tori due to computational cost and the interplay between the internal frequencies. Additionally, branches can be seen to move upwards and downwards in the subplots. It is interesting to note that we did not tell the continuation algorithm to move ”up” or ”down”. We initialized a solution in the center manifold and let the continuation algorithm go from there. Attempts to go in the ”other” direction were made, but it was found that solutions do not exist on both sides at a given halo orbit.

**Fig. 8:**
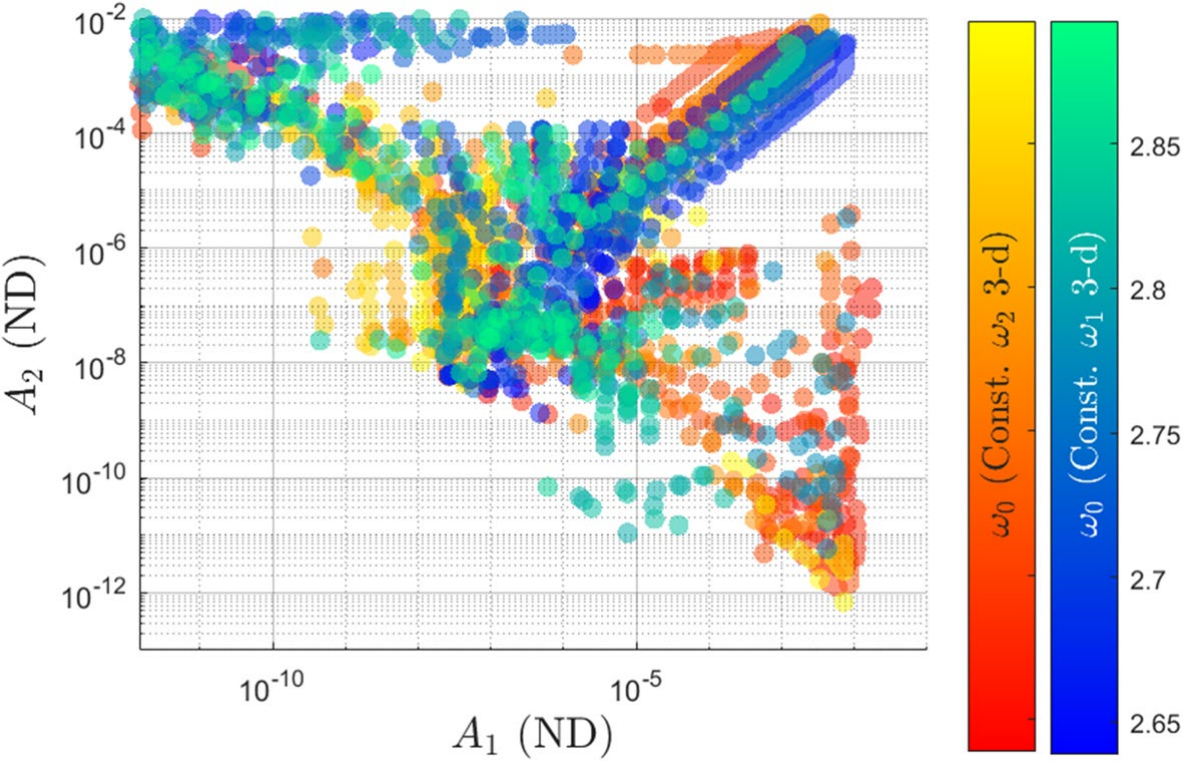
Distribution of amplitudes for the branches of 3-d quasi-halos

### Amplitudes

Earlier it was stated that $\omega _1$ corresponds to motion in CM-A and $\omega _2$ corresponds to motion in CM-B. Continuing with the notation, then $A_1$ is the amplitude of motion in CM-A and $A_2$ is the amplitude of motion in CM-B. As such the constant $\omega _2$ 2-d family has invariant surfaces with amplitudes $A_1$, the constant $\omega _1$ 2-d family has invariant curves with amplitudes $A_2$, and the constant $\omega _2$ 3-d and constant $\omega _1$ 3-d families have invariant surfaces with both amplitudes. The amplitudes for each elliptic quasi-halo family are shown in Fig. 8 in a log-log plot. The constant $\omega _2$ 3-d family adheres to the left color bar while the constant $\omega _1$ 3-d family adheres to the right color bar. The points are colored according to the $\omega _0$ value of their corresponding quasi-periodic orbit (this also identifies which halo orbit that branch originates from). The amplitudes of the initial quasi-halos for each branch are around 1e-8 in both dimensions for reference. The distribution of the amplitudes between the two families appears to be symmetric. Many of the orbits grow large in both dimensions while some of them grow small in one of the dimensions. It should be noted that at some point when one of the dimensions gets too small that orbit can no longer be considered a 3-d quasi-halo; rather it has degenerated to a 2-d quasi-halo. This degeneracy point should be the error tolerance used for convergence.

From Fig. 8 it can be seen that some of the constant $\omega _2$ 3-d family members have a large $A_1$ and a small $A_2$ (bottom right region), while few family members have large $A_2$ and small $A_1$ (top left region). This shows a preference for $A_1$ to grow for the constant $\omega _2$ 3-d family. The opposite behavior is seen for the constant $\omega _1$ 3-d family, which shows a preference for $A_2$ to grow while $A_1$ remains small. Recall that the amplitude $A_1$ is tied to the magnitude of oscillations in CM-A, while the amplitude $A_2$ is tied to the magnitude of oscillations in CM-B. Then, the observed behaviors *logically* make sense because freezing $\omega _2$ should limit the growth of the amplitude $A_2$ in CM-B. Since the initial excitement of CM-B is small, then $A_2$ *should* remain small. Likewise for freezing $\omega _1$. However, looking at the top right region of the figure, it is seen that most of the branches contain orbits which grow equally in both amplitudes. This result contradicts the logical argument presented above, so it is unknown why the amplitudes grow the way that they do in these families. Figure 8 does not present a relationship between the growth of the frequencies and the growth of the amplitudes, however this will be shown in the next section.

**Fig. 9:**
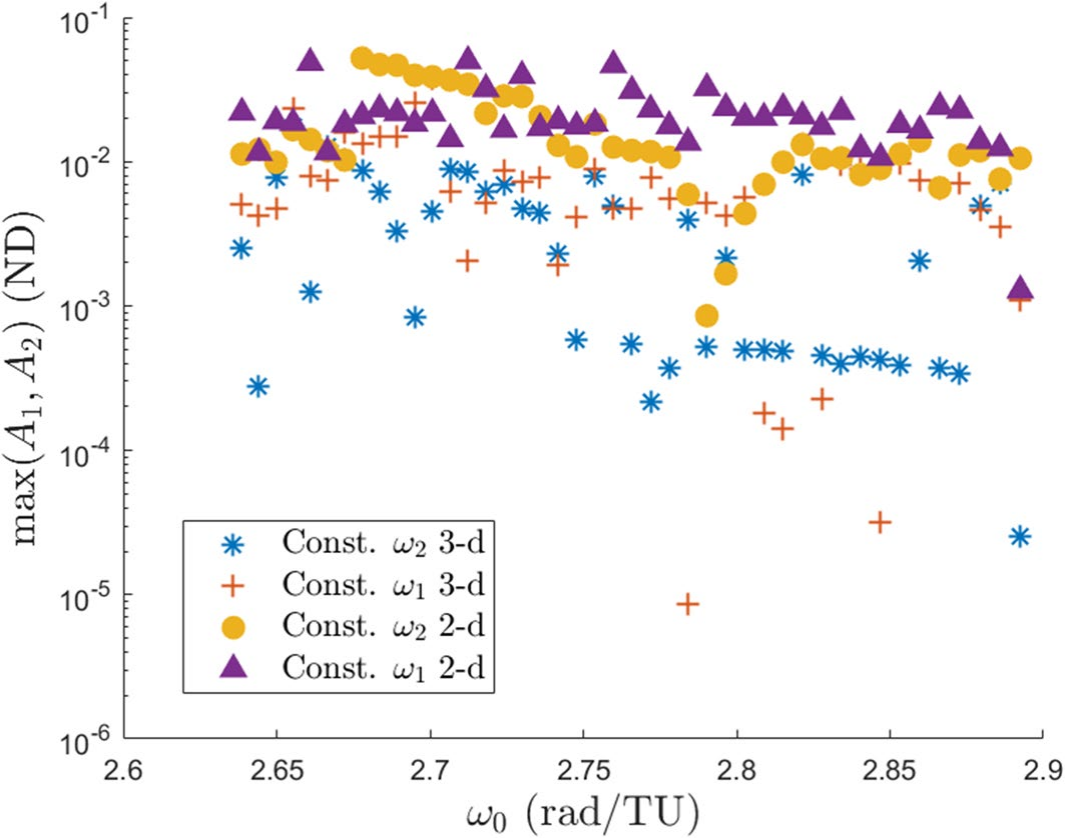
Maximum amplitudes within each of the four computed branch types

**Fig. 10:**
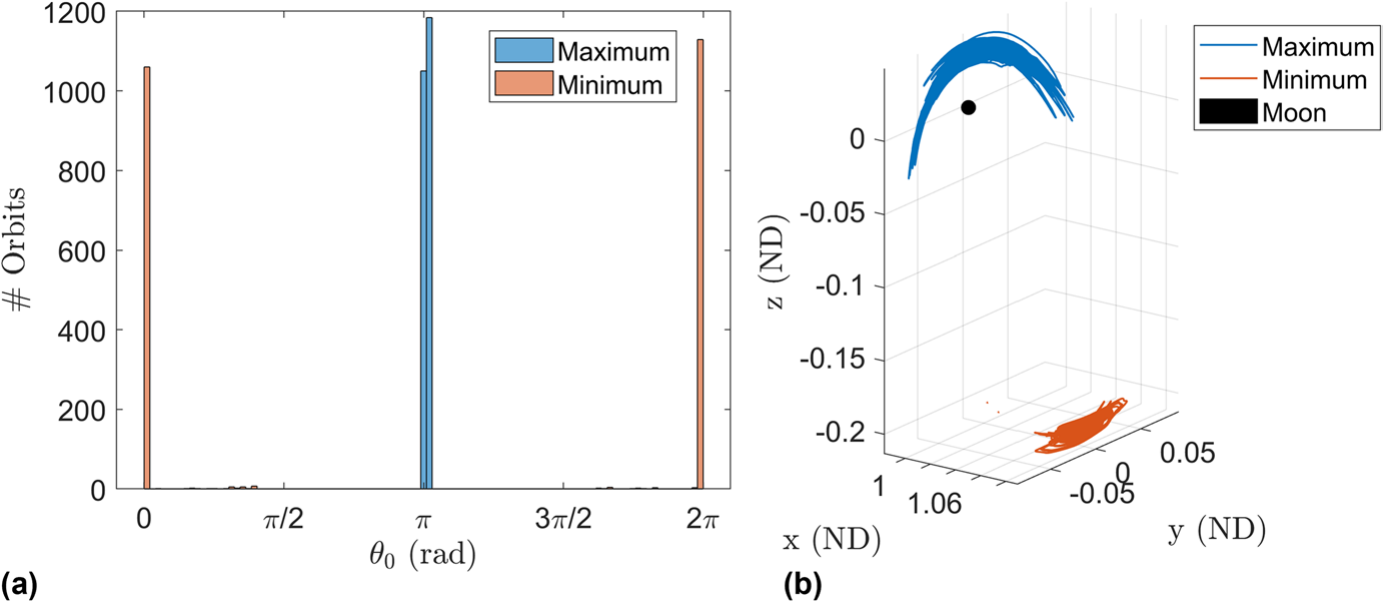
Histogram of of location where maximum and minimum amplitude along each 2-d QPO occurs (**a**) and a plot of the invariant curves in configuration space with maximum and minimum amplitude for each 2-d QPO (**b**)

A comparison is made to the amplitudes of the 2-d quasi-halos and shown in Fig. 9. The four marker types correspond to each of the four branch types while each data point is the maximum amplitude within that branch of orbits with fixed $\omega _0$. It is seen that the maximum size of the 2-d QPO branches are typically several times larger than the 3-d QPO branches. In dimensional units the maximum amplitude of: the constant $\omega _2$ 2-d family is 20,000 km, the constant $\omega _1$ 2-d family is 19,000 km, the constant $\omega _2$ 3-d family is 6700 km, and the constant $\omega _1$ 3-d family is 14,000 km. For reference the maximum amplitude of the 2-d quasi-halo orbits in [11] is 117,800 km. It should be noted that the amplitudes are the sizes of the invariant surface used in GMOS and not the average size of the surface as it moves along in the $\theta _0$ direction of the torus. It turns out there is generally a single location along the $\theta _0$ direction where the 2-d quasi-halo orbits in the vicinity of the stable halo orbits attain their maximum or minimum amplitudes, namely the point closest to the secondary ($\theta _0 = \pi$) and the point furthest from the secondary ($\theta _0 = 0$), respectively (Fig. 10).

### Jacobi Constant

**Fig. 11:**
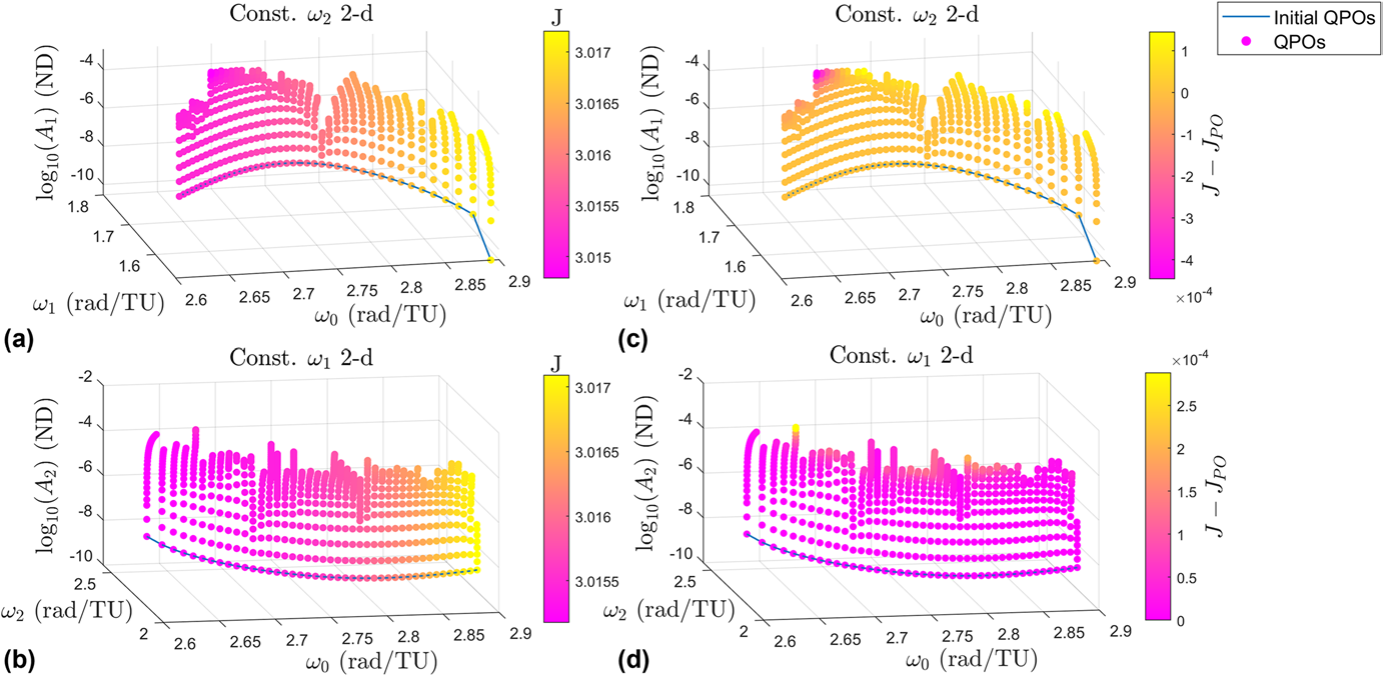
Plots displaying the Jacobi constant for the 2-d quasi-halo branches

**Fig. 12:**
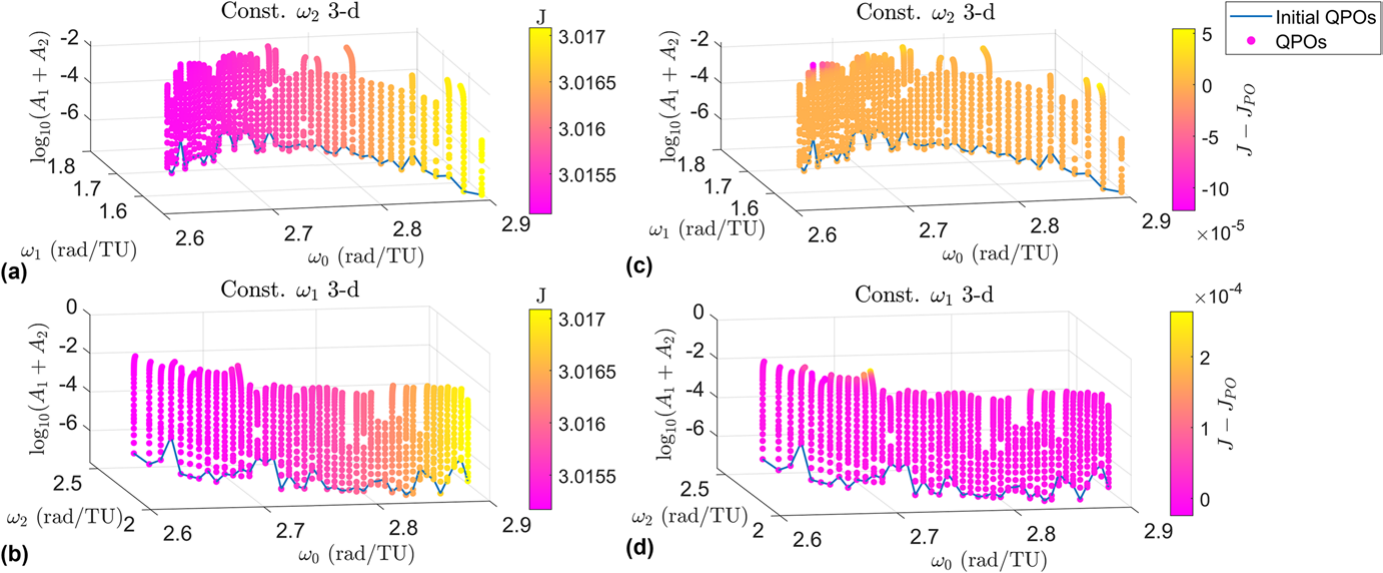
Plots displaying the Jacobi constant for the 3-d quasi-halo branches

The Jacobi constant is plotted as a color gradient for each of the two 2-d families in the left column in Fig. 11. The right column contains the same plots but with a different color gradient. This color gradient represents the change in Jacobi constant among each branch compared to the Jacobi constant of the halo orbit each branch originates from. The top row shows the constant $\omega _2$ 2-d family while the bottom row shows the constant $\omega _1$ 2-d family. Figure 12 shows the same information but shows the 3-d families instead. The plots in the left columns show that as $\omega _0$ increases the Jacobi constant increases. This is the same behavior the halo orbits themselves follow. Each branch appears to be a single color, showing there is little change in the Jacobi constant as the orbit amplitudes grow larger. The more interesting observations come from the plots in the right columns.

The first observation is that the Jacobi constant does not change very much as the orbits grow larger, however near the ends of many branches the free frequency changes more rapidly as does the Jacobi constant. This seems to suggest that rapid changes in the frequencies and the Jacobi constant compared to the sizes of the orbits indicate branches are nearing the end of the family. The second observation is that the Jacobi constant decreases along some of the branches while increasing along others. Lujan and Scheeres show in [11] the Jacobi constant typically decreases as the $L_2$ quasi-halo orbits grow larger. This is largely seen with the quasi-halos emanating from the halo orbits with type *center* x *center* x *saddle*. However, the Jacobi constant exhibits a more complex behavior in the vicinity of the stable halo orbits.

The Jacobi constants computed lie in the range [3.014788704506776, 3.017204276227027] for the constant $\omega _2$ 2-d family, [3.015176655614008, 3.017088403921683] for the constant $\omega _1$ 2-d family, [3.015065998407869, 3.017086688496275] for the constant $\omega _2$ 3-d family, and [3.015169926957731, 3.017087620465851] for the constant $\omega _1$ 3-d family.

### Stability

#### Stability Bifurcation

The elliptic quasi-halo orbits are stable, so there are no hyperbolic manifolds to utilize to approach or depart these objects. The benefit of the stability property is that small perturbations will not cause an asymptotic departure from the nominal orbit. At most there will be a bounded secular drift caused by the difference in frequencies between the nominal orbit and the orbit a spacecraft has been perturbed onto. The 2-d quasi-halos in the region come in two stability types: partially elliptic and partially hyperbolic [11]. The partially elliptic quasi-halo orbits are stable and behave similarly to the elliptic orbits, however a small perturbation will generally excite the third mode of oscillation creating a 3-d quasi-halo. This is one reason it is important to study the 3-d quasi-halo orbits. The partially hyperbolic orbits have a hyperbolic manifold emanating from them. These orbits and other partially hyperbolic quasi-halo orbits and unstable halo orbits can be utilized for low-energy transfers to the stable region. Once the spacecraft is close enough a maneuver or some other transfer design (such as in McCarthy and Howell [3]) can lead the spacecraft to a stable orbit.

**Fig. 13:**
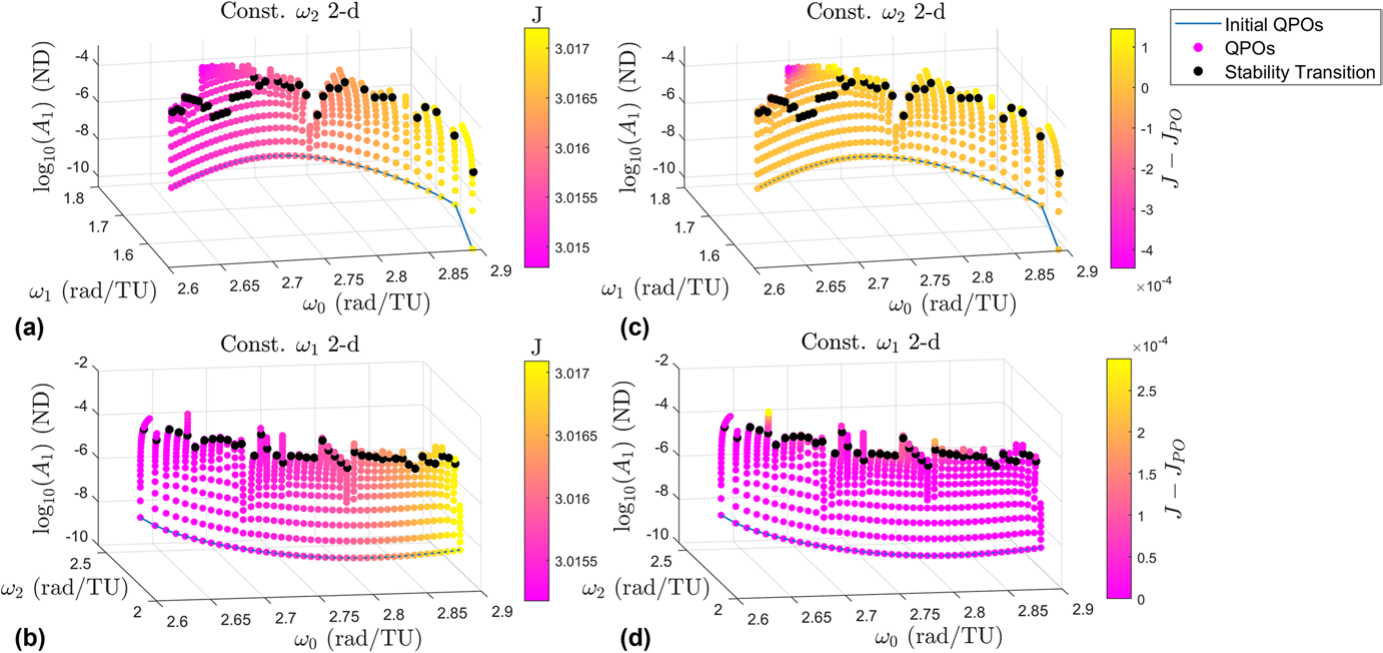
Stability transition in the 2-d quasi-halo branches

In prior work by Lujan and Scheeres [11] the stability of the 2-d quasi-halo orbits is determined by the eigenvalues of the stability matrix from GMOS [17] and sorted and categorized according to the method of Jorba [21]. This method proved to be too numerically unstable to accurately classify the stability of the orbits in the stable region of the halo orbits. In this work small deviations on the order of 1e-10 are made from the invariant curves of the 2-d quasi-halo orbits and propagated to various points in time. The propagated points are compared to the original invariant curve. This analysis is a visual inspection in order to distinguish between stable and weakly unstable behavior. After combing through each 2-d quasi-halo branch the points are identified and plotted in Fig. 13. This figure is the same as Fig. 11 but with the identified bifurcation points where the branches transition from stable to unstable.

**Fig. 14:**
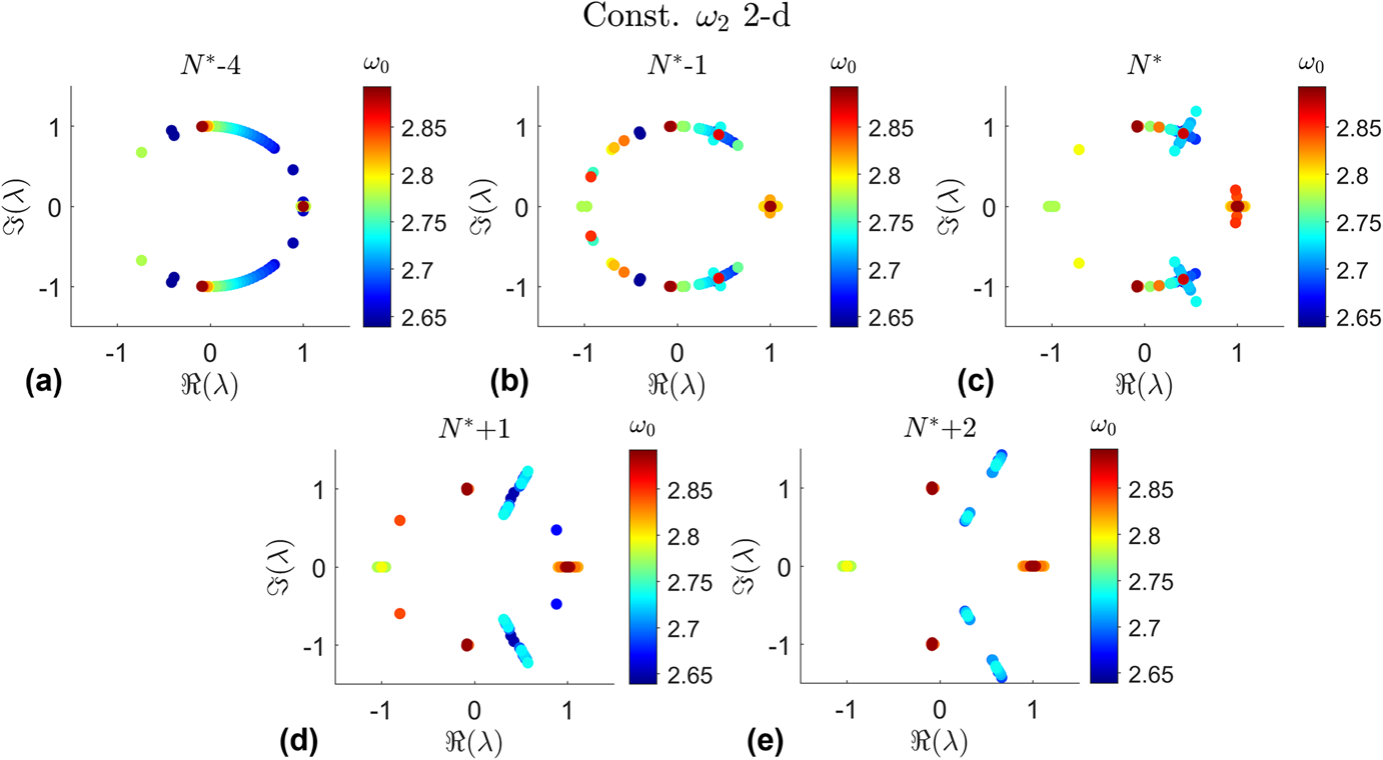
Eigenvalues near the identified bifurcation points from the visual analysis for each constant $\omega _2$ 2-d branch

**Fig. 15:**
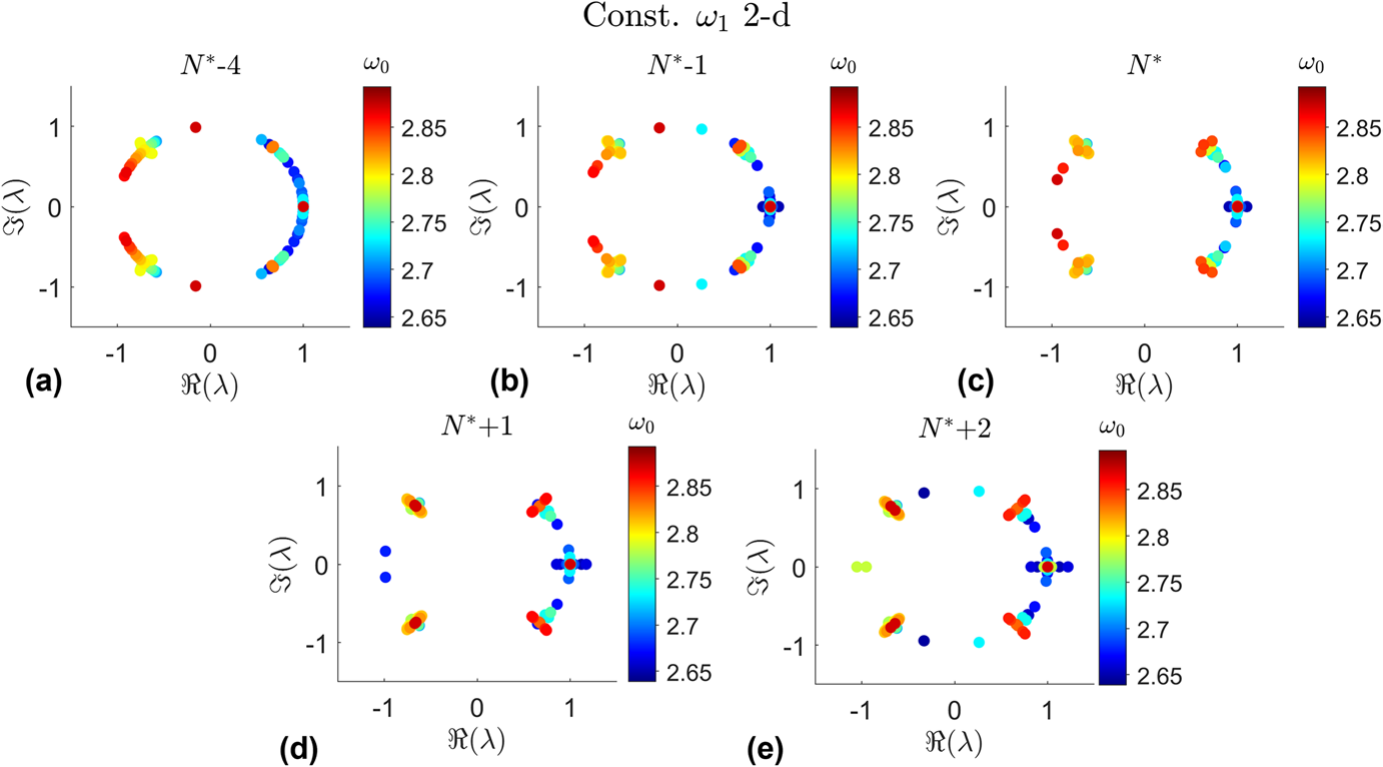
Eigenvalues near the identified bifurcation points from the visual analysis for each constant $\omega _1$ 2-d branch

The locations of these bifurcation points are generally consistent with the behavior of the eigenvalues. Figure 14 shows the eigenvalues of the quasi-halo orbits at the identified bifurcation points and for orbits before and after the bifurcation point in the continuation process for each constant $\omega _2$ 2-d branch. Likewise Fig. 15 shows the eigenvalues of the constant $\omega _1$ 2-d branches around the bifurcation points. The orbits in a branch can be numbered 1, 2, 3,... in accordance with the order in which they are computed. The number at which the last orbit is stable before becoming unstable in the visual analysis is defined as $N^*$. Then the notation $N^* \pm n$ is the $n^{th}$ orbit computed after or before the identified transition point $N^*$. Orbits before the transition point are stable while the orbits after the transition point are unstable. The color of each point in each plot is in accordance with the halo orbit from which each quasi-halo has been generated from.

What is seen in Fig. 14 is the progression of the eigenvalues as the continuation procedure approaches and passes through the bifurcation points for each constant $\omega _2$ 2-d branch. The mechanism for the bifurcations are the collisions between eigenvalues that push them off of the unit circle. It is clear that the eigenvalues begin on the unit circle, indicating stability, and break off as they near the identified stability transition point from the visual analysis. There are 3 locations where the eigenvalues break from the unit circle. The eigenvalues that collide at plus or minus 1 break off onto the real axis. The eigenvectors of these eigenvalues give the tangent directions to the stable and unstable manifolds. The eigenvalues that collide on the unit circle away from plus and minus 1 break into a complex quadruple, indicating the formation of a complex saddle. A similar behavior is seen for the constant $\omega _1$ 2-d family in Fig. 15.

In Lujan and Scheeres [11] it is conjectured that the transition points are thought to bound the sizes of the 3-d quasi-halo orbits. To test this theory the amplitudes of the partially elliptic quasi-halos are compared with the amplitudes of the elliptic quasi-halos in the same manner that Fig. 9 is constructed but with the partially hyperbolic orbits removed. This comparison revealed that there are elliptic orbits with amplitudes larger than the partially elliptic orbit amplitudes, so no conclusions can be drawn from the comparison with our data.

#### Region of Stability

All the orbits in this work are numerically computed with a pseudo-arclength continuation method which terminates when either the maximum number of family members has been computed or when the step size falls below the minimum step size. Outside of these conditions the cause of termination of the continuation method is not always certain. The termination could be due to approaching a resonance that the continuation was unable to move past or it could be due to the branch reaching the end of the family. To check for sizes of potentially missing QPOs a numerical perturbation analysis is performed. The idea behind this test is that since this region is stable, then any points perturbed from the stable halo orbits should remain in the area, given that the magnitude of the perturbation is small enough. The perturbed points that remain in the area after some amount of time should generically be on 3-d quasi-halo orbits. The size of the perturbation at which orbits begin to depart the area can be used to quantify the size of the stability region. This test will answer the question, given a perturbation magnitude from a stable halo orbit, how likely is it that the point will remain in the area (i.e. in the stable region)?

**Fig. 16:**
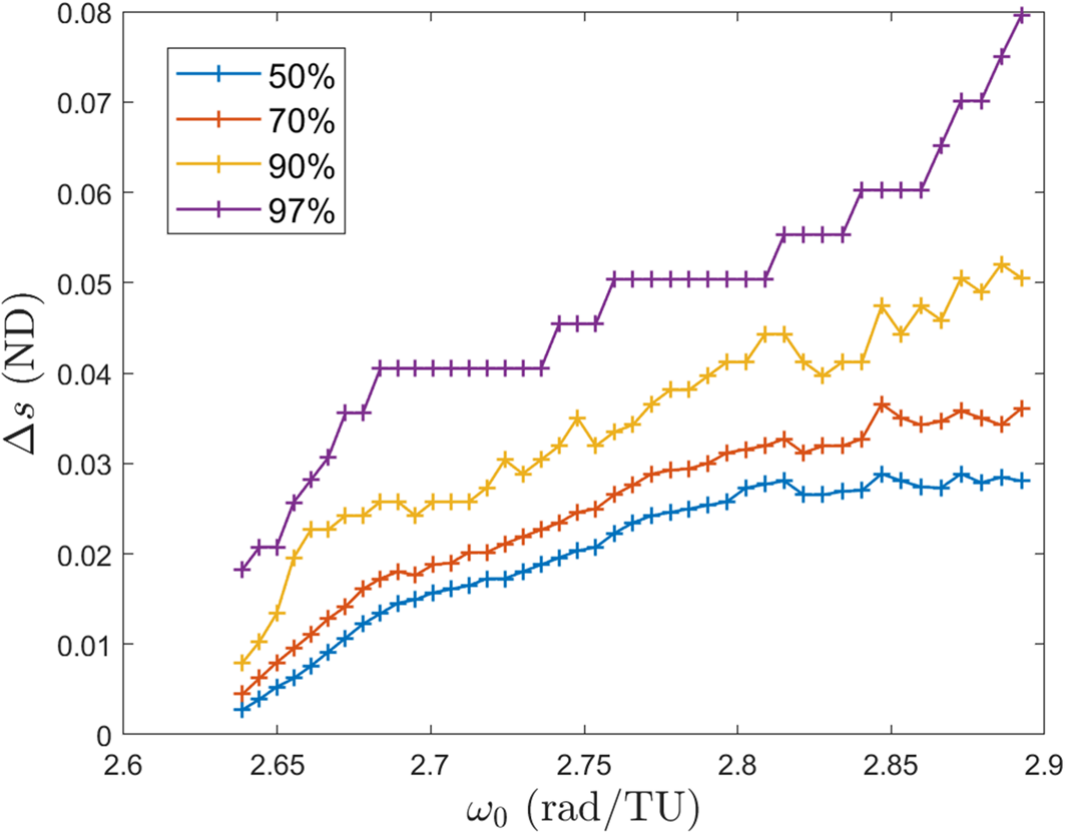
Empirical estimates of the size of the stable region surrounding the stable halo orbits

The test is performed by initializing points with a given perturbation size and then propagating them forward in time. At the end of the time they are determined to have either departed or remained in the stable region based on their distance from the underlying halo orbit. For this test 1,500 points are initialized in the center subspaces of a stable halo orbit from Eq. (4) with a particular step size $\Delta s$ and various values of $\theta _0$ and $\theta _1$ which each partition $[0,2\pi )$. Those points are then propagated out to 10 orbital periods of the periodic orbit from which they emanate from. The minimum distance from each point to the halo orbit is computed at the final time. Points with a distance of more than 1.3 times the initial step size are considered to have departed the area. The percentage of departed trajectories is then calculated. A departure percentage is targeted from a bisection method to find the step size required for that departure percentage. For a targeted departure percentage the step size is found for each stable halo orbit and recorded. The targeted departure percentages are 50%, 70%, 90%, and 97%, and the results are in Fig. 16.

**Fig. 17:**
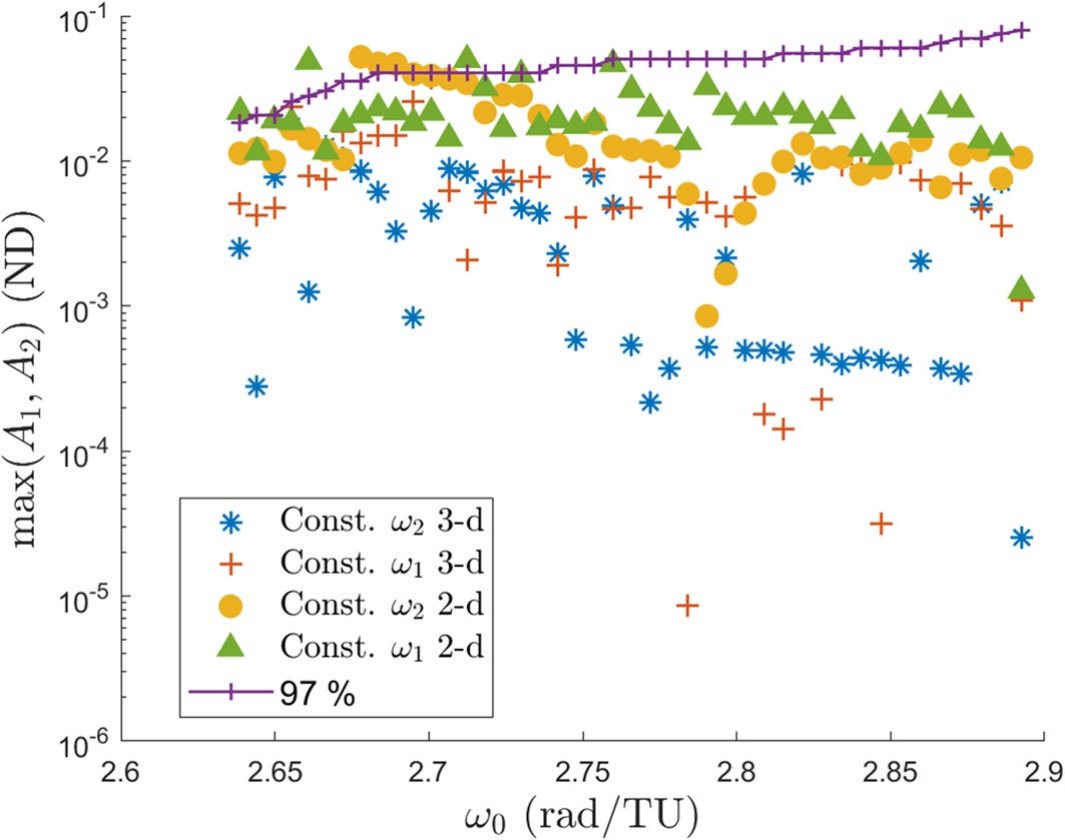
Comparison of the 97% line with the computed orbit amplitudes

An interesting result from this experiment is that as $\omega _0$ increases moving through the stable halo orbits the step size needed to reach the departure percentage continues to increase and is several times larger at the end of the halo orbits than at the beginning. At first the step size increases because the perturbed points are getting further away from the boundary between stable and unstable halo orbits. It was thought that the step sizes would decrease as the perturbed points approach the end of the stable halo orbits, however this is not observed. The general increase in the step size suggests that the hyperbolic manifold after the stable region is weaker than the hyperbolic manifold prior to the stable region. A larger step size is needed in order to push the perturbed points outside the threshold in the allotted amount of time. From this idea it can be inferred that the actual step size needed to stay in the stable region is smaller than the found step size. The hypothesis about the strength of the hyperbolic manifolds is confirmed from the Lyapunov exponents of the 2-d quasi-halo orbits emanating from outside the stable halo orbit region in Lujan and Scheeres [11].

The 97% line in Fig. 16 serves as a theoretical limit to the size of the stable region, and hence the maximum size of quasi-halo orbits in this region. This line is then compared with the amplitudes from Fig. 9 to determine if we have reasonably found the maximum sizes of quasi-halo orbits in this region. The comparison between the region of stability and the orbit amplitudes seem to agree fairly well in Fig. 17. Some branches of the 2-d quasi-halos have orbits with amplitudes larger than the step sizes that comprise the 97% line while most of the branches lie below this line. Toward the right side of the plot where $\omega _0$ is larger the gap between the orbit amplitudes and the 97% line grows. This could indicate that the 97% line should begin sloping downward to account for the weaker hyperbolic manifold as mentioned above, or it could be that the continuation procedure terminated prematurely and did not find larger orbits.

### Geometry

The 2-d quasi-halo orbits are quasi-periodic tori of dimension 2 and have invariant surfaces that are closed curves in the 6-dimensional phase space of the CR3BP. As one of these curves evolves in time it extrudes out the shape of the entire quasi-halo orbit forming a 2-dimensional surface like that in Fig. 1. When this object is projected into configuration space the object is still a surface, however it becomes self-intersecting as noted by Lujan and Scheeres in [11]. The motion of a spacecraft on a 2-d quasi-halo orbit lies on the surface of this object.

**Fig. 18:**
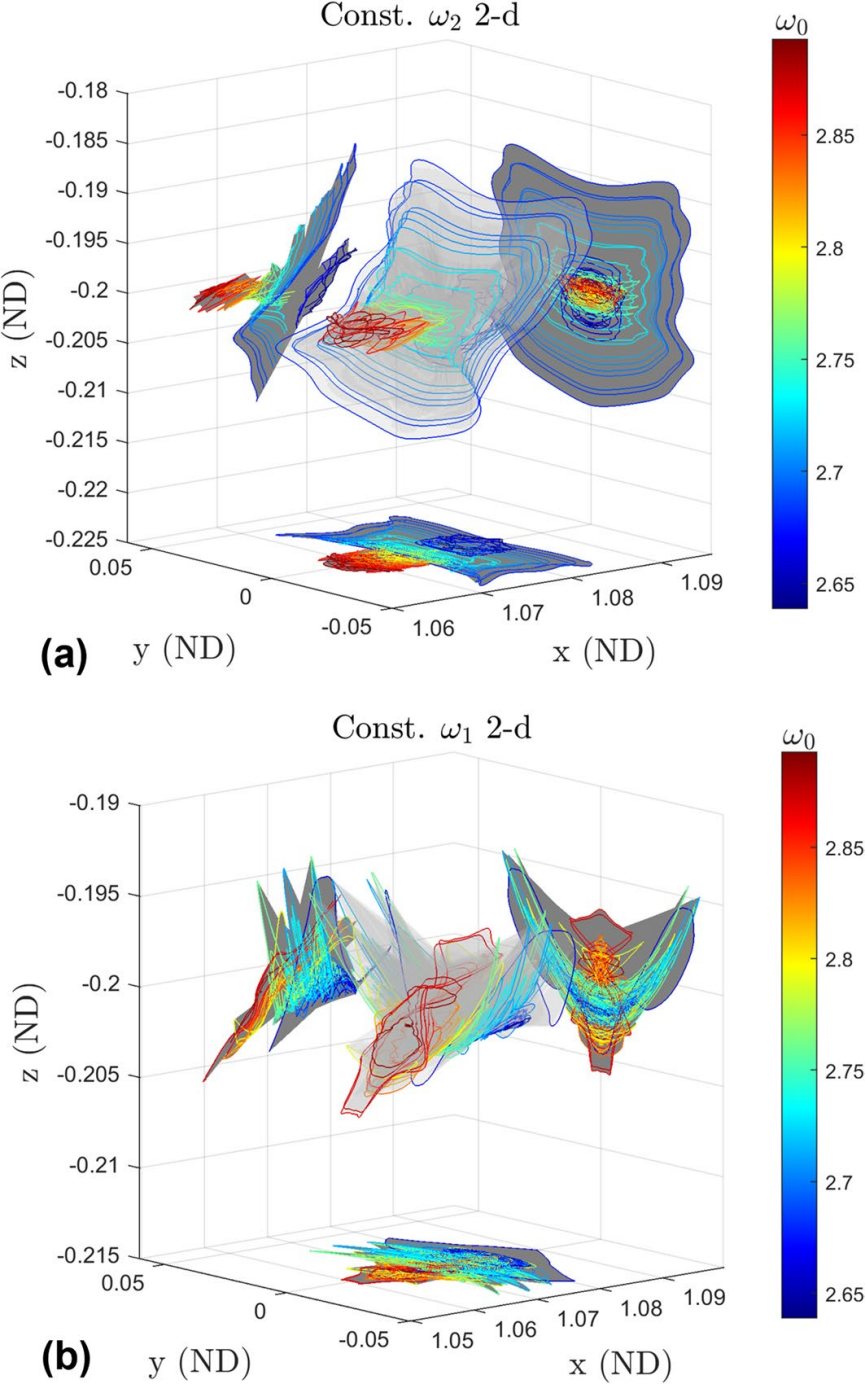
Surface (with shadows) made by the last invariant curve of each branch in the constant $\omega _2$ 2-d family (a) and in the constant $\omega _1$ 2-d family (b)

While we cannot show all of the 2-d quasi-halo orbits on a single plot we use the invariant curves to construct a surface encapsulating all of the invariant curves computed in GMOS for each of the two corresponding branch types. This is done by taking the last invariant curve from each branch of a given branch type and constructing a surface that connects the curves to each other. The surface created in this fashion for the constant $\omega _2$ 2-d family is in plot (a) of Fig. 18 while the surface created in this fashion for the constant $\omega _1$ 2-d family is in plot (b). The invariant curves used to construct each surface are plotted and colored according to the value of $\omega _0$ they possess. Coloring them this way allows one to see how the invariant curves change as the underlying stable halo orbit changes.

The sharp changes in the surface are a result of the differences in size and shape between the last invariant curve from one branch to another. These differences arise from the partitioning of the stable halo orbits, from dynamical effects from crossing over and getting stuck by resonances, and the numerical reasons of termination of the program.

The 3-d quasi-halo orbits are quasi-periodic tori of dimension 3 and have invariant surfaces that are 2-dimensional surfaces in the 6-dimensional phase space of the CR3BP. The extrusion of one of these surfaces in time forms a 3-dimensional surface which fills a volume in any 3-dimensional subspace of the 6-dimensional phase space. The fact that the orbit fills a volume in configuration space makes it difficult to view and understand the motion of a spacecraft on one of these orbits. Additionally, the method of constructing a Poincaré map of a constant Jacobi energy family (such as in [9]) cannot be applied to view and analyze the elliptical quasi-halo orbits. However, looking at the invariant surfaces individually in configuration space gives geometrical insight into the full orbits.

**Fig. 19:**
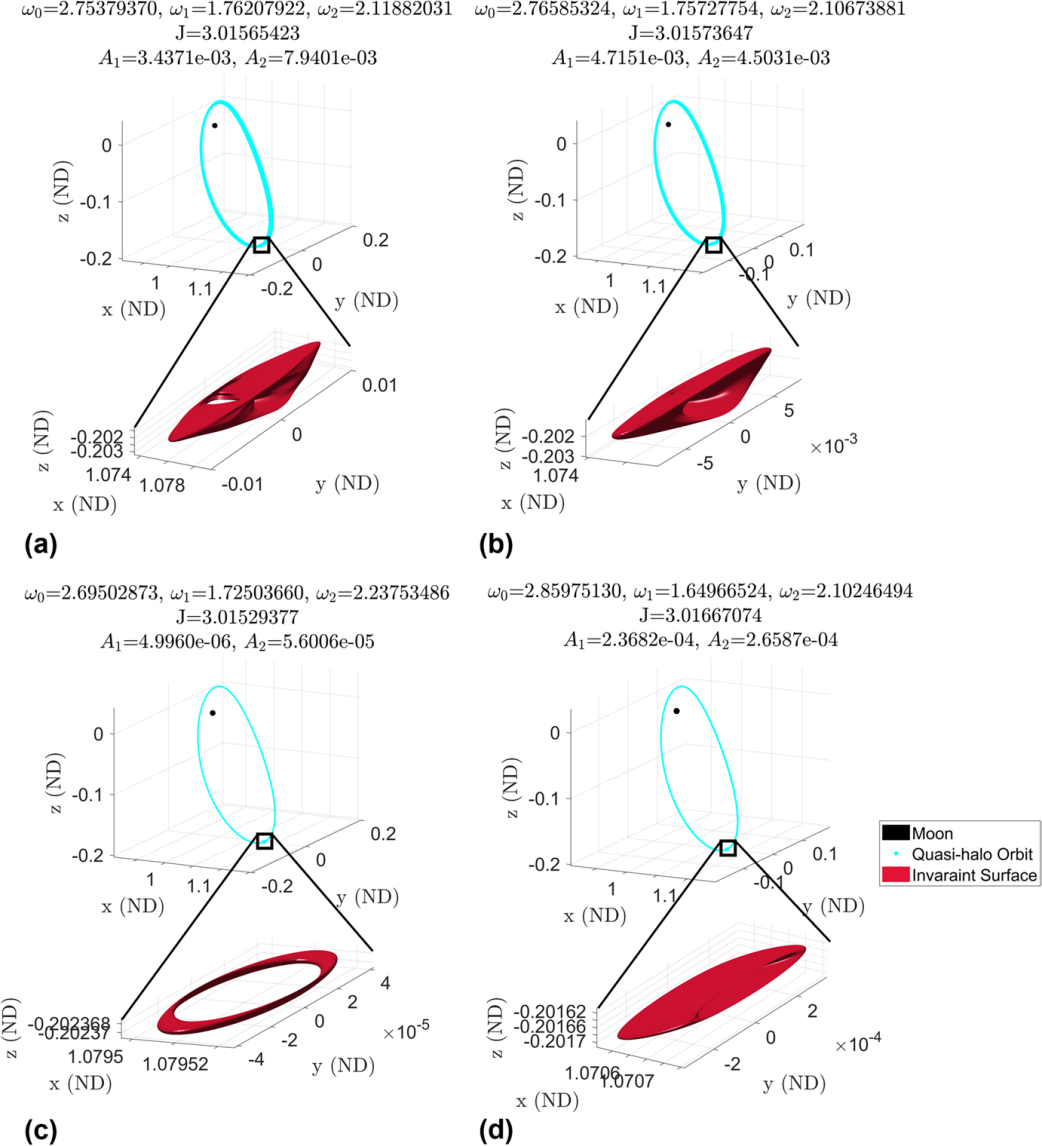
Examples of elliptic quasi-halo orbits in configuration space

Figure 19 shows four examples of elliptic quasi-halo orbits in configuration space along with the Moon for a reference on size. Additionally, the invariant surfaces computed in GMOS for each orbit are shown. Comparing these orbit amplitudes to Figs. 8 and 9 shows that plots (a) and (b) are among the largest of the elliptical quasi-halo orbits while plots (c) and (d) are among medium-sized orbits. This figure shows just how small the elliptic quasi-halo orbits are compared to the partially hyperbolic orbits in Figs. 10 and 23 of Lujan and Scheeres [11].

**Fig. 20:**
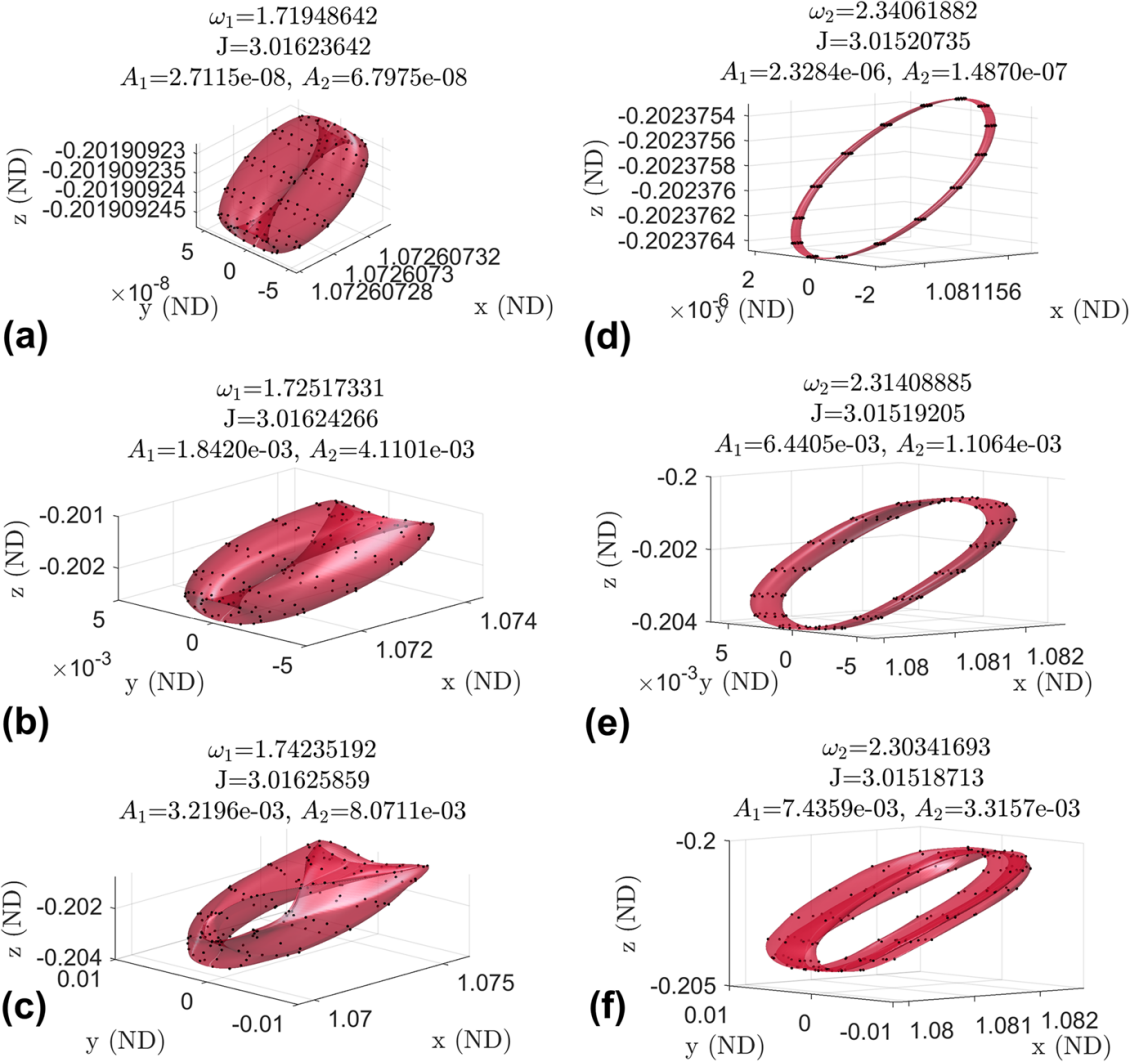
Invariant surfaces within a constant $\omega _2$ 3-d branch (a-c) and within a constant $\omega _1$ 3-d branch (d-f)

**Fig. 21:**
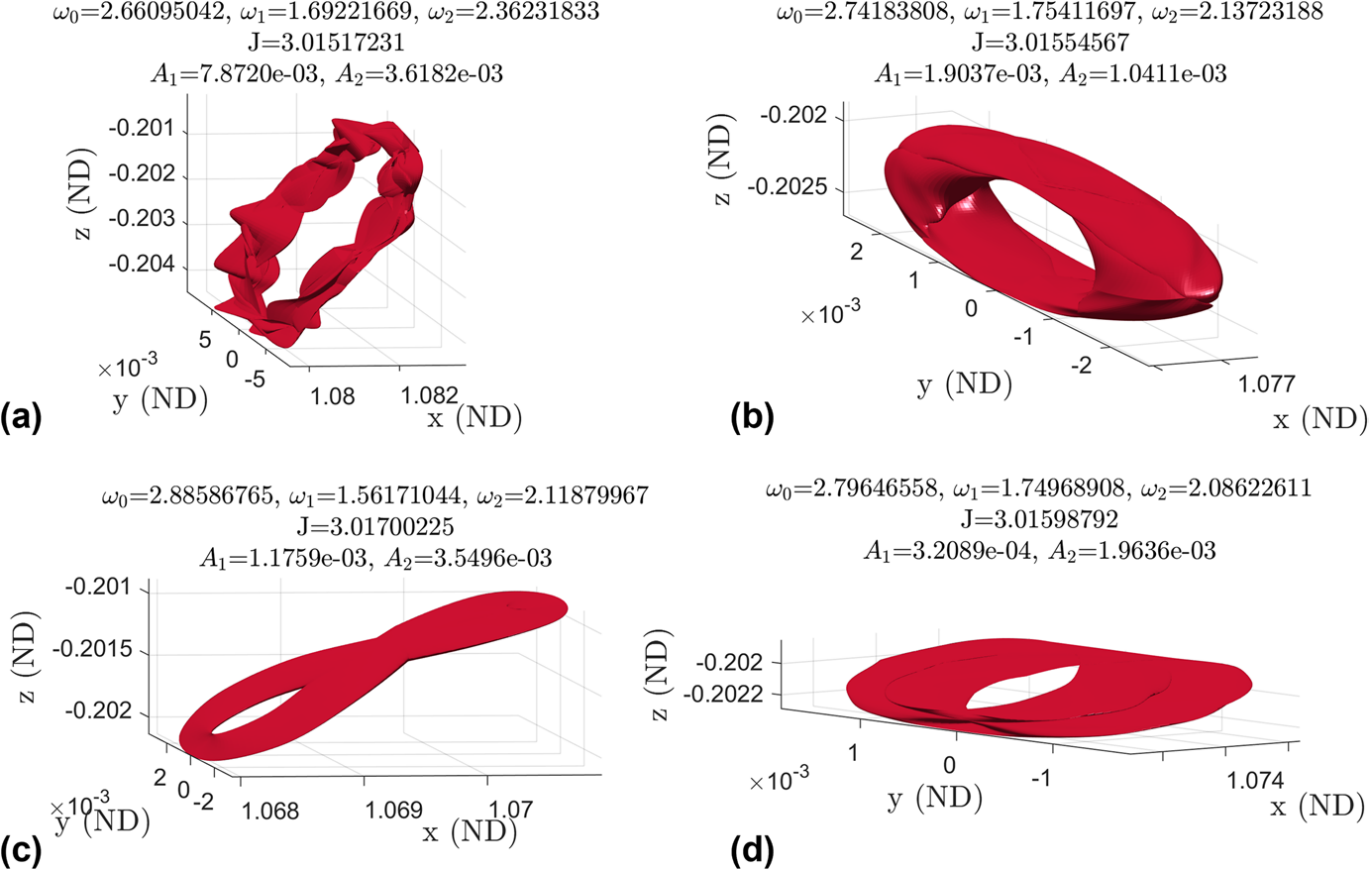
Survey of invariant surfaces in the elliptic quasi-halo family

Figure 20 shows the growth and evolution in the continuation procedure of the invariant surface for a constant $\omega _2$ 3-d branch and a constant $\omega _1$ 3-d branch. In each plot is the points representing the surface from GMOS and the interpolated surface. A constant $\omega _2$ 3-d branch is in plots (a), (b), and (c). This branch has constant frequencies $\omega _0 = 2.821444$ and $\omega _2 = 2.085261$. Plot (a) is taken near the beginning of the branch, plot (b) is taken from the middle of the branch, and plot (c) is taken toward the end of the branch. Plots (d), (e), and (f) shows the growth and evolution of the invariant surface for a constant $\omega _1$ 3-d branch. This branch has constant frequencies $\omega _0 = 2.666571$ and $\omega _1 = 1.698082$. Plot (d) is taken near the beginning of the branch, plot (e) is taken from the middle of the branch, and plot (f) is taken toward the end of the branch. The constant $\omega _2$ 3-d branch has an increase in Jacobi constant along the branch, while the constant $\omega _1$ 3-d branch has a decrease in Jacobi constant. Figure 21 shows other individual invariant surfaces we found interesting.

It should be noted that most of the invariant surfaces of the 3-d quasi-halo orbits are found to be self-intersecting, and thus the entire orbit is comprised of trajectories constantly crossing over each other. This observation calls for care and detailed analysis when placing multiple spacecraft on one of these elliptical quasi-halo orbits so as to ensure a collision will not occur. However, in light of this, the abundance of intersections means there are boundless opportunities to change the phasing on the orbit.

### Relative Motion

The static images of the invariant surfaces give insight into what the orbits look like, however they do not provide information about the motion of the invariant surface in time. To show this behavior the points constructing the invariant surfaces of 415 quasi-halo orbits have been propagated for three stroboscopic times and the relative states with respect to their underlying halo orbit have been examined.

**Fig. 22:**
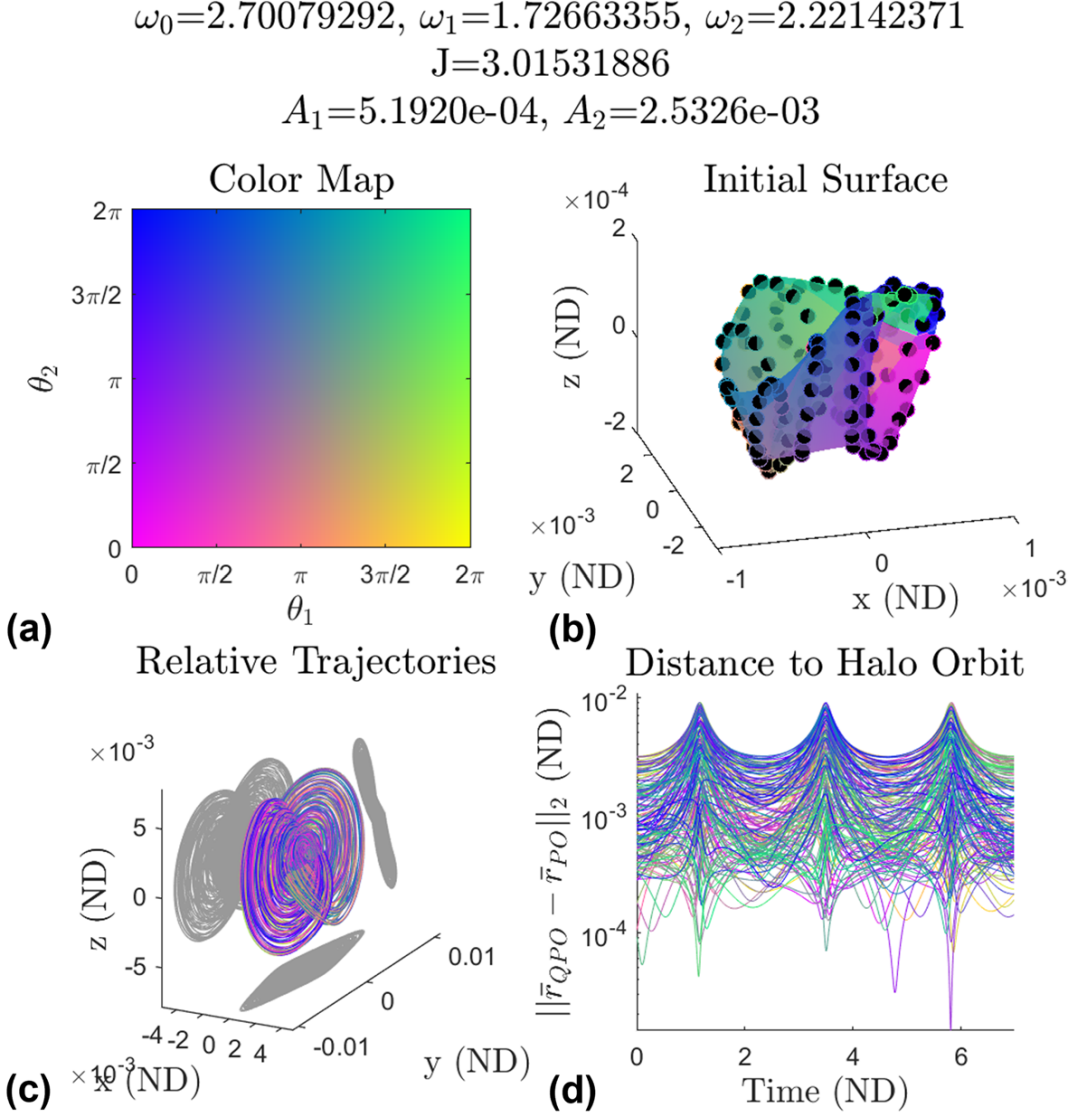
Representative example of the relative motion for the 3-d quasi-halo orbits

Figure 22 shows one example that captures the typical behavior of the relative motion. Plot (a) shows the color map used to identify each point on the invariant surface. Recall from Eq. (4) that $\theta _1$ and $\theta _2$ are angles parameterizing the surface. Each coordinate pair $(\theta _1,\theta _2)$ has a unique color. Plot (b) shows the invariant surface colored according to the color map. Plot (c) shows in configuration space the relative motion about the stable halo orbit which the orbit was generated from. Plot (d) shows the time history of the distance to the halo orbit for each point.

The initial invariant surface of plot (b) is embedded in the relative motion in plot (c). The results in plot (c) show that the invariant surface gets warped and stretched in the *y* and *z* directions while not much stretching occurs in the *x* direction. The plot also shows that there is full coverage of the halo orbit by the invariant surface. This shows that these orbits are good to place surveillance satellites to keep watch on an object on the underlying halo orbit.

Combining the information of plot (c) and plot (d) it is surmised that at half the stroboscopic time the maximal stretching occurs while also achieving its closest approach to the halo orbit meaning the invariant surface is largest at this point in time. The smallest spread in distances occurs when the invariant surface is furthest from the Moon meaning it assumes its smallest size. The point in time where the largest spread in distance occurs is when the invariant surface is making its closest approach to the Moon. And similarly the point in time where the smallest spread in distance occurs is when the invariant surface is furthest from the Moon. This behavior is seen among most of the tested elliptic quasi-halo orbits, and it coincides with where the invariant curves of the 2-d quasi-halo orbits in this region achieve their largest and smallest amplitudes (refer to Fig. 10).

Plot (d) shows that the distance to the halo orbit remains positive. This result shows that there exists a ball with a radius of about 1e-5 for which the invariant surface does not penetrate. So spacecraft on this invariant surface will not collide with a spacecraft on the underlying halo orbit. However, if there is a displacement along the $\theta _0$ direction between the invariant surface and a point on the halo orbit, then the analysis will have to be repeated to ensure there are no crossings with the underlying halo orbit. It is shown in Lujan and Scheeres [11] that the underlying halo orbits usually penetrate the surfaces of the 2-d quasi-halo orbits, so it is reasonable to assume that the underlying stable halo orbits penetrate the elliptic quasi-halo orbits. The difference between the penetrations is that for the 2-d quasi-halo orbits there are a finite number of penetrations since a line is crossing through an infinitely thin surface. However, the elliptic quasi-halo orbits fill a volume, so there would be an infinite number of penetrations along the crossings.

**Fig. 23:**
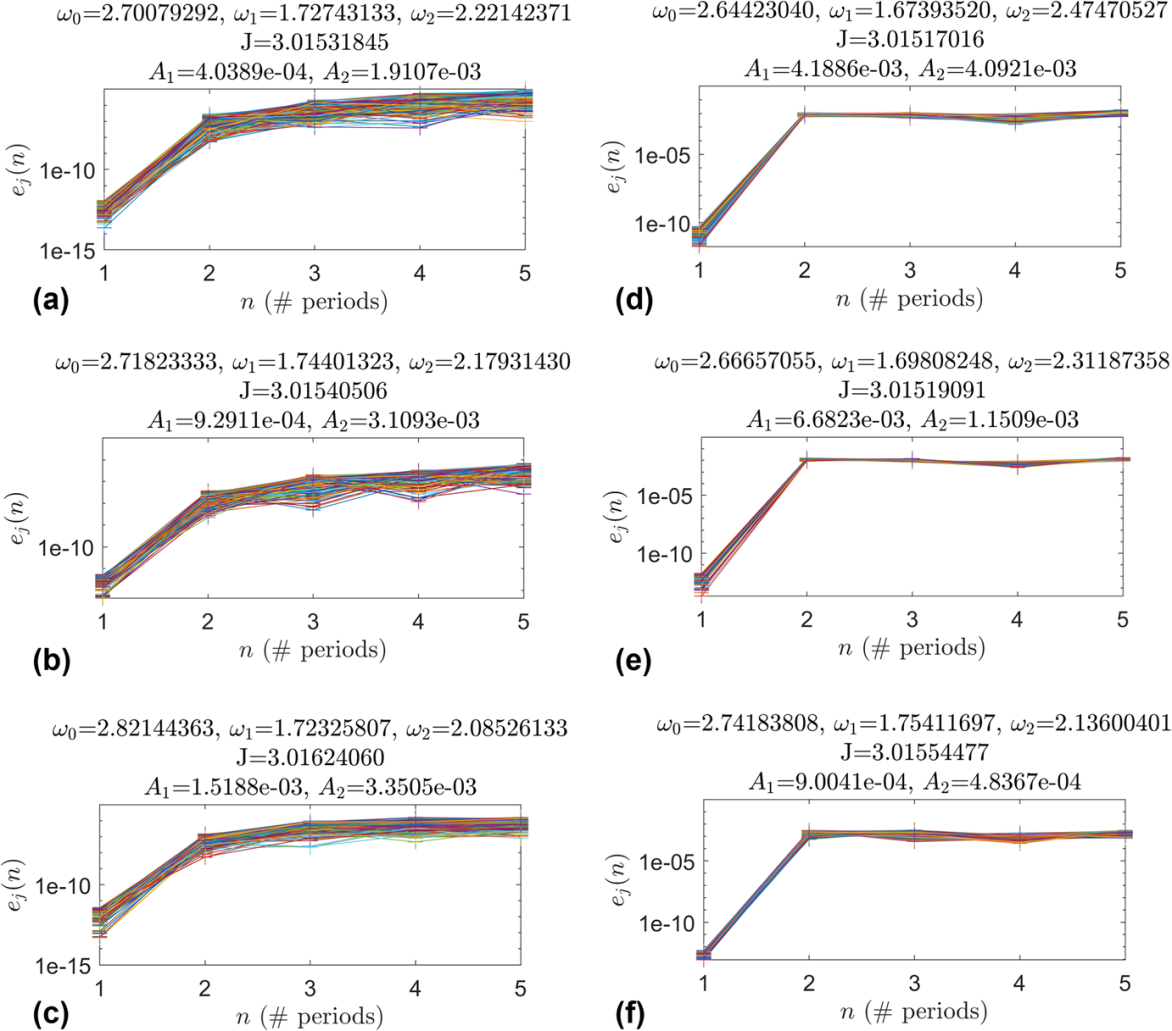
The error over time between six computed invariant surfaces from GMOS and the propagated surfaces

### Error Analysis

As mentioned in Sect. 5 the tolerance level used to compute all of the invariant surfaces and curves in this work is 7e-11. Since each point incurs some amount of error then each point is actually on a different quasi-periodic torus with different frequencies. This means there is a difference between the actual and idealized orbits of the discrete points representing an invariant surface from GMOS. To measure this difference the points ${\varvec{X}}$ of an elliptic quasi-halo invariant surface from GMOS are propagated forward with the stroboscopic map $x\mapsto \phi _T(x)$. The points $\phi _T({\varvec{X}})$ are rotated by the rotation matrix $R_{-{\varvec{\rho }}}$ to get the states of where the points should be on the invariant surface. The states should coincide with ${\varvec{X}}$, so the error is the norm of the difference between these quantities. The points $\phi _T({\varvec{X}})$ are then mapped to $\phi _{2T}({\varvec{X}})$ and rotated by the rotation matrix $R_{-2{\varvec{\rho }}}$. The error is calculated as the norm of the difference between the newly rotated points and the original points ${\varvec{X}}$. Recall that the vector ${\varvec{X}}$ is a vector comprised of points in phase space ${\varvec{x}}_j$ that represent points on the invariant surface of a QPO. Then the equation for the error for point ${\varvec{x}}_j$ is given in Eq. (13). This process is repeated for a total of five mappings and the results for six different quasi-halo orbits are given in Fig. 23.

13 $$\begin{aligned} e_j(n) = ||R_{-n{\varvec{\rho }}}\phi _{nT}({\varvec{x}}_j)-{\varvec{x}}_j|| \end{aligned}$$

In Eq. (13) when $n=1$ then this is equivalent to the quasi-periodicity constraint of Eq. (5) divided by the number of points representing the surface. When $n=1$ the error in Fig. 23 is below the error tolerance 7e-11, showing that GMOS is indeed satisfying the quasi-periodicity constraint. However as ${\varvec{X}}$ is mapped further in time the errors grow, showing that the actual orbits diverge form the idealized orbits. In plots (a), (b), and (c) the errors grow to about 1e-8 after two stroboscopic maps and steadily grow for the following mappings. These orbits are from constant $\omega _2$ 3-d branches. In plots (d), (e), and (f) the errors grow to about 1e-2 after two stroboscopic mappings and stay nearly constant for the following mappings. These orbits are from constant $\omega _1$ 3-d branches.

## Conclusions

This work uses a single-parameter continuation method called GMOS to explore the dynamical structure in the vicinity of the $L_2$ stable halo orbits in the Earth-Moon system of the circular restricted three-body problem. The types of solutions explored are the quasi-halo orbits diffeomorphic to 2- and 3-dimensional quasi-periodic tori. Two branches from each types of orbits are computed from the span of stable halo orbits to construct a total of four 2-parameter families. The four branch types are the constant $\omega _2$ 2-d branch, the constant $\omega _1$ 2-d branch, the constant $\omega _2$ 3-d branch, and the constant $\omega _1$ 3-d branch. In each branch a parameter is held constant and the value of that parameter is determined by the halo orbit and its monodromy matrix from which the branch is grown.

The 3-d quasi-halo orbits do not have amplitudes as large as the 2-d quasi-halo orbits, but are larger than some of the partially elliptic quasi-halo orbits. However, all of the orbits in this region are much smaller than the quasi-halo orbits emanating from the unstable quasi-halo orbits. The amplitudes of all the orbits are compared to an empirical limit to the sizes of orbits in this region.

The Jacobi constant among each of the four branch types has a net change in the range [-4e-4 3e-4] compared to the value of the Jacobi constant of the underlying halo orbit from which each branch is generated. The direction of change has a turning point within the constant $\omega _2$ 2-d and constant $\omega _2$ 3-d families. The branches with smaller $\omega _0$ values have a negative change in the Jacobi constant as orbits grow larger. And branches with larger $\omega _0$ values have a positive change in the Jacobi constant as orbits grow larger. The constant $\omega _1$ 2-d and constant $\omega _1$ 3-d branches exhibit an increase in the Jacobi constant as the orbits grow larger.

The 2-d quasi-halo orbits are comprised of partially elliptic and partially hyperbolic quasi-halo orbits. Nearly each branch is identified to have a stability bifurcation leading from stable to unstable orbits. All identified bifurcation points are near the ends of branches. The partially hyperbolic orbits do not transition back to partially elliptic before the end of the branch is reached.

Lastly, the geometry of the invariant surfaces of these quasi-halo orbits are diverse. The invariant surfaces of the elliptic quasi-halos create a volume in phase space when evolved in time resulting in a trajectory with complex behavior. The relative motion of the invariant surfaces with respect to the underlying halo orbit provides full coverage of a point on the halo orbit with the same phasing in $\theta _0$.
